# Biomarker potential of plasma cell-free DNA for cholangiocarcinoma

**DOI:** 10.1016/j.heliyon.2024.e41008

**Published:** 2024-12-06

**Authors:** Sattrachai Prasopdee, Sissades Tongsima, Montinee Pholhelm, Siraphatsorn Yusuk, Sithichoke Tangphatsornruang, Kritiya Butthongkomvong, Teva Phanaksri, Anthicha Kunjantarachot, Jutharat Kulsantiwong, Smarn Tesana, Thanakrit Sathavornmanee, Veerachai Thitapakorn

**Affiliations:** a Research Group in Multidimensional Health and Disease (MHD), Chulabhorn International College of Medicine, Thammasat University, Pathum Thani, 12120, Thailand; b Thammasat Research Unit in Opisthorchiasis, Cholangiocarcinoma, and Neglected Parasitic Diseases, Thammasat University, Pathum Thani, 12120, Thailand; c Chulabhorn International College of Medicine, Thammasat University, Pathum Thani, 12120, Thailand; d National Biobank of Thailand (NBT), National Science and Technology Development Agency, Pathum Thani, 12120, Thailand; e National Center for Genetic Engineering and Biotechnology, National Science and Technology Development Agency, Pathum Thani, 12120, Thailand; f Medical Oncology Unit, Udonthani Cancer Hospital, Udon Thani, 41330, Thailand; g Faculty of Science, Udon Thani Rajabhat University, Udon Thani, 41000, Thailand; h Chonburi Hospital, Ministry of Public Health, Chonburi, 20000, Thailand

**Keywords:** Cell-free DNA, Circulating tumor DNA, Ultra-low-pass whole-genome sequencing (ULP-WGS), iChorCNA, *Opisthorchis viverrini*, Cholangiocarcinoma, Diagnosis

## Abstract

**Background:**

To prevent the development of cholangiocarcinoma, an effective screening opisthorchiasis viverrini and/or differential diagnosis of and the cholangiocarcinoma is crucial needed. The level and quality of cfDNA in plasma are being investigated for their potential role as biomarkers in cholangiocarcinoma.

**Methods:**

The study enrolled 43 healthy controls (N), 36 *O. viverrini*-infected subjects (OV), and 36 cholangiocarcinoma patients (CCA). Plasma cfDNA was quantified by fluorometry (Qubit 4), and qualified analysis, including % tumor fraction, ctDNA, ploidy number, and somatic copy number alteration (SCNA), was conducted using ULP-WGS and analyzed by iChorCNA. The statistical analysis and comparison among the groups were performed.

**Results:**

The results showed that cfDNA could effectively differentiate between N and OV from CCA statistically both by DNA amount and quality. Using a cut-off of >20.94 ng/ml, the sensitivity and specificity of the cfDNA concentration were determined to be 86.11% and 98.73% for the differential diagnosis of cholangiocarcinoma, respectively. The ULP-WGS with iChorCNA indicated the % tumor fraction of cfDNA (*P* < 0.001) and the values of the ploidy number (*P* < 0.001) of the cholangiocarcinoma group and the other groups were statistically significant. Moreover, the SCNA of the cholangiocarcinoma group was shown to be significantly high in comparison to that of the healthy control group with an odds ratio of 11.688 (*P* < 0.001).

**Conclusion:**

The use of cfDNA concentration and ULP-WGS for analyzing DNA quality including % tumor fraction, ctDNA concentration, tumor ploidy, and SCNA are useful for the differential diagnosis of cholangiocarcinoma from opisthorchiasis viverrini and healthy individuals.

## Highlights

• These findings investigated both quantitative and qualitative detection of plasma cell-free DNA in a cost-effective manner which allowing patients to access the test and potentially prevent the development of cholangiocarcinoma.
• ULP-WGS determined the SCNA in cholangiocarcinoma, revealing a pattern of chromosome 6 loss which belong the HLA locus.
• The 94.44 % sensitivity and 100 % specificity were obtained when using a cut-off of >19.78 ng/ml for cfDNA concentration.

## Graphical abstract

**Figure:**
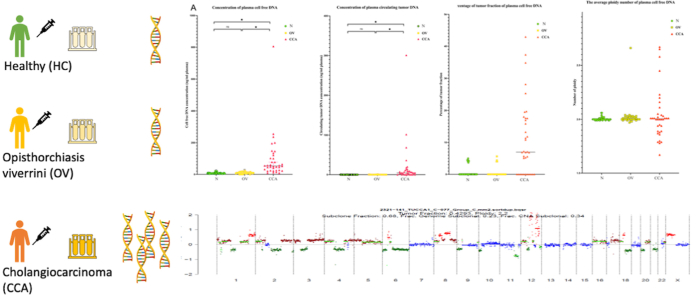

## 1 Introduction

Detection of early-stage cancer is a promising method that affects the treatment of the type of cancer. Nevertheless, effective screening or diagnostic methods are limited in several cancers. The gold standard for cancer diagnosis is histopathology of resected or biopsied tissue, which is an invasive procedure. Liquid biopsies, such as serum, plasma, tear, and saliva, are non-invasive and attractive alternatives that can be used for diagnosis and prognosis. Generally, tumor markers such as CA19-9, CA125, CEA, AFP, CA 15-3, PSA, NSE, and beta-HCG are used to screen for cancers in combination with imaging (X-ray, ultrasonography, CT, MRI, and PET scan). The overall method is limited and cannot effectively detect early-stage cancer. Since next-generation sequencing (NGS) has been launched and widely used, cell-free DNA (cfDNA), circulating tumor DNA (ctDNA), circulating miRNA, exosomal miRNA, and lncRNA have been intensively investigated for their role in diagnoses, prognoses, and therapeutic approaches. Interestingly, cfDNA has been widely used for the detection of genetic diseases through non-invasive prenatal testing. It was later reported that the application of cfDNA in cancer can detect leukemias 10 years before the onset of the illness [1].

Cancer is known as a disease that accumulates mutation. The mutations such as point mutation, frameshift, deletion, insertion, and amplification commonly occur in the cancer cell genome. However, investigation of genome alteration has been commonly analyzed by tissue-based exome sequencing, which is an invasive sample. Liquid biopsy samples such as blood, serum, plasma, urine, and saliva are alternative sources of non-invasive samples, which were previously limited for their diagnostic potential. This allows the detection of genome alteration of cancer-based liquid biopsies such as cfDNA or ctDNA.

Cholangiocarcinoma (CCA), an epithelial bile duct cancer, is a type of cancer with high morbidity and mortality. Most of the diagnosed patients have late-stage cancer, which is the result of the non-availability of an effective diagnostic method. The discovery of a novel biomarker for diagnosing CCA has been intensively done and is in the pipeline. The most important risk factor of CCA is the combination of the *Opisthorchis viverrini* infection and a nitrosamine. The inflammation and the nitrosamine damage the DNA of cholangiocytes and consequently mutate and develop CCA. Biomarkers that can identify the transition of opisthorchiasis to CCA are urgently needed. Once opisthorchiasis is detected in a patient, treatment with praziquantel is highly effective. This prevents the development of CCA. Recently, powerful biomarker, cfDNA or ctDNA, has been applied. The amount of cfDNA in patients’ blood circulation was found to have increased in several cancers and inflammations. The non-apoptotic DNA fragments were released from the dead cells and could be detected by a high sensitivity method. Normally, the cfDNA in the blood circulation is less than 50 ng/ml [2], while a higher concentration of cfDNA may indicate abnormal homeostasis or diseases including cancers. Moreover, the increase, methylation, and mutation of cfDNA or ctDNA in plasma or serum indicates the presence of cancers [[3], [4], [5], [6]]. Therefore, concentration measurement and/or detection of genome alteration by using plasma cfDNA may be helpful. The application of whole-genome sequencing in clinical approaches is limited regarding the cost of sequencing. Recently, Adalsteinsson and colleagues reported a significant method called ultra-low-pass whole-genome sequencing (ULP-WGS) and iChorCNA tools for WGS [7]. Regarding the low coverage sequencing, this tool is particularly useful in terms of saving time and cost-effectiveness with high efficacy in CCA [8]. Therefore, in this study, quantitative analysis of cfDNA level in plasma, alongside qualitative analysis using the ULP-WGS method and the iChorCNA tool, was selected, implemented, and tested for its efficacy in diagnostic liquid biopsy approaches for cholangiocarcinoma (CCA).

## 2 Materials and methods

### 2.1 Chemicals and reagents

EDTA blood tubes were purchased from Becton Dickinson. The MagMAX™ Cell-Free DNA Isolation Kit for plasma cell-free DNA extraction was purchased from Applied Biosystem™, Thermo Fisher Scientific. The measurement of DNA concentration was done by using Qubit dsDNA HS. The DNA library was constructed by Ion Plus Fragment Library Kit (Applied Biosystem™, Thermo Fisher Scientific).

### 2.2 Subjects

This study was approved by the Human Ethics Committee of Udonthani Cancer Hospital, Ministry of Public Health, Thailand (Project No. UCH-CT 11/2563). In reference to *O. viverrini*, liver fluke infection is a risk factor for cholangiocarcinoma (CCA). *O.* *viverrini-*infected patients were also included in this study. The participants were categorized into three distinct groups based on their respective health conditions. The study included three groups: the healthy control group (N), the *O. viverrini*-infected subject group (OV), and the cholangiocarcinoma (CCA) subject group. For the N subjects, physical examination revealed no abnormalities, including no liver enlargement, jaundice, or positive results for *O. viverrini* eggs in the fecal examination. Individuals who tested positive for *O. viverrini* eggs in fecal examinations, despite normal liver and bile duct structures as determined by ultrasonography, were enrolled in the OV group. Notably, all participants in the OV group exhibited no symptoms. For the CCA group, enrollment was determined by a definitive diagnosis made through histopathology. The number of plasma samples included in this study was 43, 36, and 36 from the N, OV, and CCA groups, respectively. The demographic and clinical information of the subjects is summarized in Table 1.

**Table 1:** Summary of subject demography.

	Group		
	N	OV	CCA
**Sample size**	43	36	36
**Sex (Male/Female)**	21/22	19/17	19/17
**Age (year)**			
Min/Max	21/60	18/79	32/79
Mean ± SD	38.7 ± 9.8	55.8 ± 14.4	58.6 ± 9.9
**Alcohol consumption**			
No	21 (49%)	11 (31%)	15 (42%)
Yes	22 (51%)	25 (69%)	21 (58%)
**Smoking**			
No	32 (74%)	21 (58%)	21 (58%)
Yes	11 (26%)	15 (42%)	15 (42%)
**Raw fish eating-habit (Source of *O. viverrini* infection)**			
No	26 (60%)	3 (8%)	6 (17%)
Yes	17 (40%)	33 (92%)	29 (80%)
Uncertain	0 (0%)	0 (0%)	1 (3%)
**History of *O. viverrini* infection**			
No	42 (98%)	33 (92%)	27 (75%)
Yes	1 (2%)	3 (8%)	8 (22%)
Uncertain	0 (0%)	0 (0%)	1 (3%)
**Fermented food eating-habit (Source of nitrosamine)**			
No	2 (5%)	0 (0%)	0 (0%)
Yes	41 (95%)	36 (100%)	35 (97%)
Uncertain	0 (0%)	0 (0%)	1 (3%)
**Cancer stage**			
Stage 1 (%)	0 (0%)	0 (0%)	1 (3%)
Stage 2 (%)	0 (0%)	0 (0%)	0 (0%)
Stage 3 (%)	0 (0%)	0 (0%)	1 (3%)
Stage 4 (%)	0 (0%)	0 (0%)	34 (94%)

### 2.3 Blood collection and plasma preparation

The 10 ml venous blood samples were collected, and EDTA was utilized as an anticoagulant. The tube was subsequently centrifuged at 3000×*g* for 10 min. The supernatant was transferred to a fresh tube and centrifuged again at 15,000×*g* for 10 min. The 700 μl plasma (supernatant) was aliquoted into a new tube to avoid the freeze-thaw cycle and was stored at −80 °C until further use. The turbid, hemolyzed, and clotted plasmas were excluded from this study.

### 2.4 Cell-free DNA isolation and concentration measurement

The MagMAX™ Cell-Free DNA Isolation Kit (Thermo Fisher Scientific) was used for isolating cfDNA from the 600 μl plasma of each subject. The isolated cfDNA was eluted in 35 μl eluent. Two microliters of each cfDNA eluate were used for concentration measurement by using Qubit dsDNA HS (Thermo Fisher Scientific). The concentrations of cfDNA were calculated to ng of cfDNA per 1 ml of plasma unit (ng/ml plasma). This cfDNA was then used for library construction. The ctDNA concentration was calculated from the cfDNA concentration and obtained as a percent of tumor fraction, as follows:

ctDNA = (cfDNA x % tumor fraction)/100

### 2.5 Qualification of DNA by ultra-low-pass whole-genome sequencing (ULP-WGS)

The isolated cfDNA was utilized to construct a cfDNA library in accordance with the supplied protocol (Ion Plus Fragment Library Kit, Applied Biosystems™, Thermo Fisher Scientific). Subsequently, the library constructs were subjected to sequencing utilizing the MGISEQ-2000 sequencing system (MGI Tech Co. Ltd., China). The percentage of tumor fraction and somatic copy number alterations (SCNA), including amplifications, gains, deletions, and average ploidy number, were analyzed by ULP-WGS (0.1-0.3x genome-wide coverage). Additionally, the analysis was achieved through iChorCNA software (available for download at https://doi.org/10.1038/s41467-017-00965-y) [9].

### 2.6 Statistical and diagnostic analyses

Statistical analyses were performed using SPSS Version 26.0 (Armonk, NY: IBM Corp.). To reduce the skewness of the original data, continuous data were added with a constant of 1 to all values, and thereafter, the natural log transformed. A normal Q-Q plot was used to confirm the normality of the data. The data are normally distributed if they lie approximately on a straight diagonal line. To find the statistically significant difference in plasma cfDNA concentration, plasma ctDNA concentration, tumor fraction of plasma cfDNA, and the values of the ploidy number (difference in values calculated either as “2 - x” when x is less than or equal to 2, or “x – 2” when x is greater than 2) between the three health status groups, a one-way analysis of variance (ANOVA) and a subsequent post hoc analysis were used if the data proved to be a normal distribution. An F-test followed by a Scheffe post hoc test was used if variances were equal across groups. A Welch test followed by Dunnett's C post hoc test was used if variances were not equal across groups. If the data were not normally distributed, the Kruskal-Wallis test would be used instead to analyze the data, followed by a Dunn's pairwise comparison. To find the association between SCNA and health status, Pearson's chi-squared test was used. If the *P* value of the Pearson's chi-squared test was <0.05, then the data were analyzed using binary logistic regression. The dot plot and the area under the receiver operating characteristics curve (AUC) were analyzed using GraphPad Prism 9. The maximum likelihood ratio was employed to determine the diagnostic threshold. The cut-off where the specificity reached 100% was also calculated. Diagnostic parameters, including sensitivity, specificity, positive predictive value (PPV), negative predictive value (NPV), and accuracy, were calculated utilizing MedCalc, a diagnostic test evaluation calculator (https://www.medcalc.org/calc/diagnostic_test.php).

## 3 Results

### 3.1 Statistical tests determination

The cfDNA concentrations in the three health status groups were statistically analyzed with an ANOVA Welch test followed by a Dunnett's C post hoc test. The statistical evidence of a difference in plasma ctDNA concentration, tumor fraction of plasma cfDNA, and difference in values of the ploidy number between the health status groups were obtained by a Kruskal-Wallis analysis.

### 3.2 Increment of plasma cfDNA concentration in CCA

**Fig. 1:**
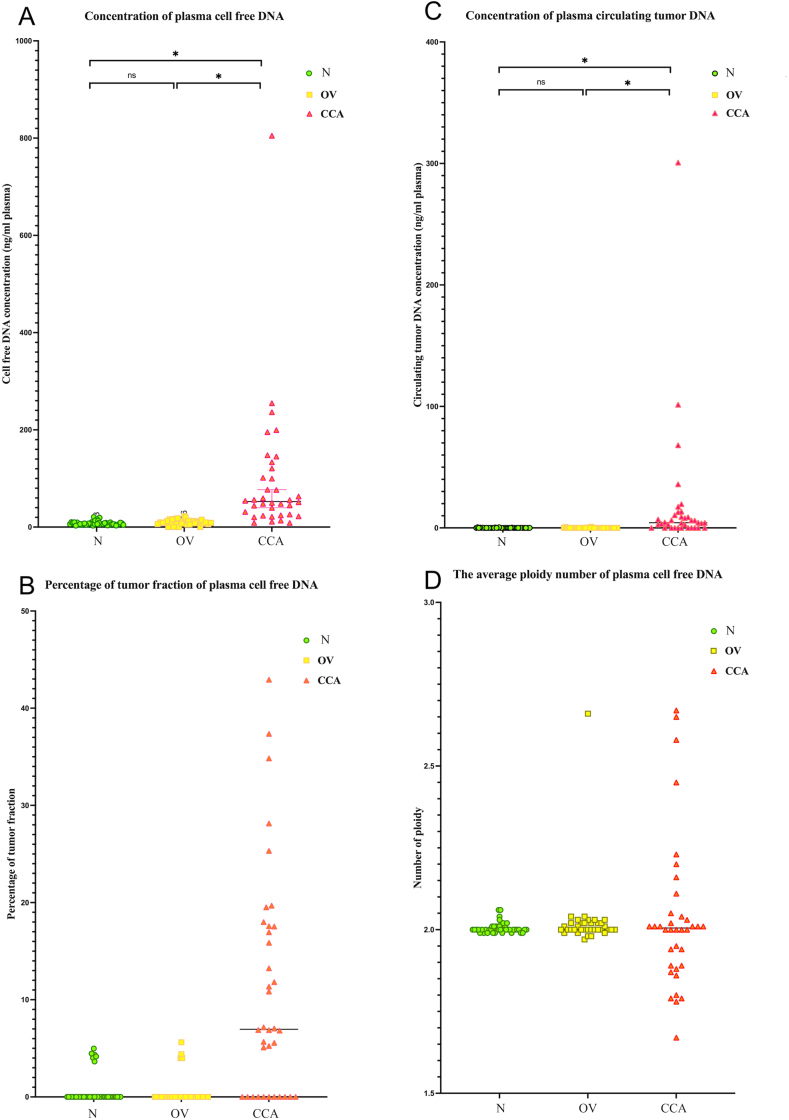
The scatter plot of plasma cell-free DNA (cfDNA) concentration (A), percentage of tumor fraction (B), circulating tumor DNA (ctDNA) concentration (C), and average ploidy number (D). The plasma cell-free DNA (cfDNA) concentration, circulating tumor (ctDNA) concentration, percentage of tumor fraction, and average ploidy number is on the y-axis and group is on the x-axis. The green circle, the yellow square, and the pink triangle indicate the N, OV, and CCA respectively.

**Fig. 2:**
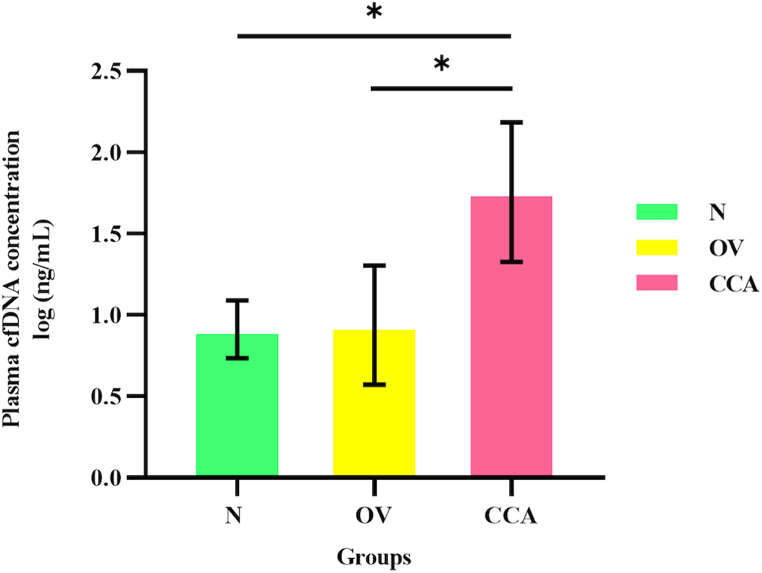
Bar chart of the plasma cfDNA concentration. The log plasma cfDNA concentration (ng/ml plasma) and group are plotted on the y- and x-axis, respectively. The error bars indicate the standard deviation of plasma cfDNA concentration. The asterisk (∗) indicates the statistical significance *P* < 0.05 of N vs CCA and OV vs CCA.

**Fig. 3:**
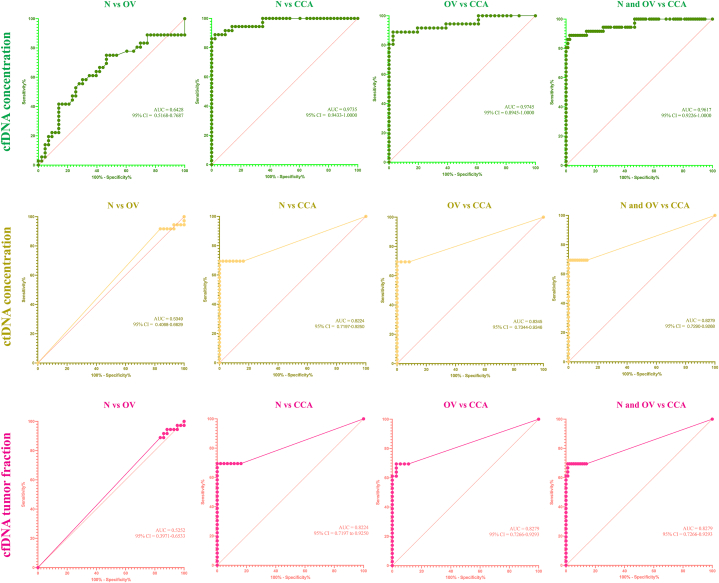
The AUC of the ROC curve of plasma cell-free DNA (cfDNA) concentration, circulating tumor DNA (ctDNA) concentration, tumor fraction. The % sensitivity is plotted against 100% - % specificity on the X-axis and Y-axis, respectively. The AUC of ROC curves and 95% CI are calculated and indicated in each curve.

The cfDNA concentrations (Fig. 1A) and the iChorCNA results, including tumor fraction (Fig. 1B), ctDNA concentration (Fig. 1C), average ploidy number (Fig. 1D), and SCNA of plasma cfDNA, are presented in Table 2a (N), 2b (OV), and 2c (CCA). The descriptive statistics of each group are summarized in Table 3. The average and the range (max–min ng/ml) of the cfDNA concentrations were highest in the CCA group (average = 94.950, range = 8.633–805.000) when compared to the N group (average = 7.879, range = 2.917–20.708) and the OV group (average = 9.826, range = 0.000–22.810) (Table 3). A Welch's ANOVA revealed a statistically significant difference in plasma cfDNA concentrations between at least two groups (Welch F [2, 59.181] = 60.929, *P* < 0.001). A Dunnett's C test for multiple comparisons found that the mean values of the plasma cfDNA concentrations were significantly different between the N group and the CCA group (*P* < 0.05, 95% CI: 0.657, 1.031), and between the OV and CCA groups (*P* < 0.05, 95% CI: 0.588, 1.048) (Fig. 2). There was no statistically significant difference between the N and OV groups (*P* > 0.05) (Fig. 2). The diagnostic parameters are summarized in Table 4. In a differential diagnosis of the CCA group from the OV and N groups, 80.56% sensitivity, 100% specificity, 100% PPV, 91.86% NPV, and 93.91% accuracy were obtained by using 23.16 ng/ml of plasma as a cut-off (Table 5). The AUC of the ROC curve of the CCA group vs the N group was 0.9735 (Fig. 3) while that of the CCA group vs the OV group was 0.9745 (Fig. 3). In the differential diagnosis of the CCA group from the N and OV groups, the AUC of the ROC curve was 0.9617 (Fig. 3). Although the level of plasma cfDNA concentration showed promising results in the differential diagnosis of the CCA group, the AUC of the ROC curve showed limited differentiation between the N and OV groups (0.6428) (Fig. 3).

**Table 2a:** Summarized results of cell-free DNA and iChorCNA of N group (43 samples).

Sample ID	Cell-free DNA concentration (ng/ml plasma)	Circulating tumor DNA concentration (ng/ml plasma)	Tumor fraction (%)	SCNAs	Loss (Chromosome no.)	Gain (Chromosome no.)	Amplification (Chromosome no.)	Average ploidy number
A012	6.650	0.000	0.000	No	No	No	No	1.99
A013	4.142	0.000	0.000	No	No	No	No	2.01
A014	2.917	0.000	4.202	No	No	No	No	2.01
A015	4.317	0.000	0.000	No	No	No	No	1.99
A036	3.442	0.000	0.000	No	No	No	No	2.00
A037	20.708	0.000	0.000	No	No	No	No	2.00
A038	6.125	0.000	0.000	No	No	No	No	2.00
A039	8.808	0.000	0.000	No	No	No	No	2.00
A040	3.967	0.000	0.000	No	No	No	No	2.00
A041	19.192	0.199	0.000	No	No	No	No	2.00
A042	6.358	0.478	0.000	No	No	No	No	2.00
A043	4.725	0.000	0.000	No	No	No	No	2.00
A044	9.800	0.000	0.000	No	No	No	No	2.01
A045	15.983	0.367	0.000	No	No	No	No	2.00
A046	6.475	0.000	0.000	No	No	No	No	1.99
A047	5.658	0.000	0.000	No	No	No	No	2.00
A048	6.242	0.000	0.000	No	No	No	No	2.02
A049	10.267	0.000	0.000	No	No	No	No	2.02
A050	5.600	0.000	4.342	No	No	No	No	2.06
A077	10.150	0.000	0.000	No	No	No	No	1.99
A078	6.358	0.000	0.000	No	No	No	No	2.00
A079	9.158	0.243	4.004	No	No	No	No	2.06
A080	12.950	0.000	0.000	No	No	No	No	2.00
A081	6.008	0.219	0.000	No	No	No	No	1.99
A082	6.417	0.000	0.000	No	No	No	No	2.00
A083	13.708	0.000	0.000	No	No	No	No	2.01
A084	4.783	0.000	4.162	No	No	No	No	2.04
A085	9.625	0.000	4.979	No	No	No	No	1.99
A086	9.858	0.123	0.000	No	No	No	No	2.00
A087	7.817	0.000	0.000	No	No	No	No	2.03
A088	7.467	0.000	0.000	No	No	No	No	1.99
A089	3.208	0.000	0.000	No	No	No	No	2.00
A090	7.583	0.000	0.000	No	No	No	No	2.00
A091	4.900	0.000	4.468	No	No	No	No	2.01
A092	8.342	0.000	0.000	No	No	No	No	2.02
A093	13.125	0.479	3.641	No	No	No	No	2.00
A094	8.925	0.000	0.000	No	No	No	No	1.99
A095	6.358	0.000	0.000	No	No	No	No	2.01
A096	9.217	0.000	0.000	No	No	No	No	1.99
A097	6.242	0.000	0.000	No	No	No	No	2.00
A098	3.033	0.000	0.000	No	No	No	No	2.00
A099	7.659	0.000	0.000	No	No	No	No	2.00
A100	4.550	0.000	0.000	No	No	No	No	2.00

**Table 2b:** Summarized results of cell-free DNA and iChorCNA of OV group (36 samples).

Sample ID	Cell-free DNA concentration (ng/ml plasma)	Circulating tumor DNA concentration (ng/ml plasma)	Tumor fraction (%)	SCNAs	Loss (Chromosome no.)	Gain (Chromosome no.)	Amplification (Chromosome no.)	Average tumour ploidy
B63	0.000	0.000	0.000	No	No	No	No	2.02
B66	8.983	0.000	0.000	No	No	No	No	2.00
B69	12.250	0.000	0.000	No	No	No	No	2.02
B70	9.683	0.307	0.000	No	No	No	No	2.02
B71	7.700	0.000	3.989	Yes	No	17,19	No	2.00
B72	12.717	0.000	0.000	No	No	No	No	2.00
B74	9.450	0.000	0.000	No	No	No	No	2.01
B78	11.492	0.000	0.000	No	No	No	No	2.02
B81	6.358	0.000	0.000	No	No	No	No	2.02
B83	22.808	0.000	0.000	No	No	No	No	1.99
B85	0.000	0.000	4.401	No	No	No	No	2.04
B86	0.000	0.000	0.000	No	No	No	No	2.00
B89	7.000	0.000	0.000	No	No	No	No	2.01
B96	5.892	0.912	0.000	No	No	No	No	2.03
B97	16.217	0.000	5.623	Yes	5	1-15, 17, 21, 22	No	2.66
B64	17.150	0.000	0.000	No	No	No	No	1.97
B65	9.217	0.000	0.000	No	No	No	No	2.00
B68	7.467	0.000	0.000	No	No	No	No	2.00
B73	12.483	0.000	0.000	No	No	No	No	2.01
B75	20.067	0.000	0.000	No	No	No	No	1.99
B76	6.125	0.000	0.000	No	No	No	No	2.00
B77	12.075	0.617	0.000	No	No	No	No	2.00
B79	15.400	0.000	4.005	Yes	No	17,19	No	2.03
B80	7.117	0.000	0.000	No	No	No	No	2.03
B82	15.692	0.000	0.000	No	No	No	No	2.02
B84	10.383	0.000	0.000	No	No	No	No	2.00
B87	0.000	0.000	0.000	No	No	No	No	2.03
B88	9.275	0.000	0.000	No	No	No	No	1.99
B90	12.425	0.000	0.000	No	No	No	No	2.00
B91	5.308	0.000	0.000	No	No	No	No	2.00
B92	13.300	0.000	0.000	No	No	No	No	2.00
B93	5.308	0.000	0.000	No	No	No	No	2.04
B94	7.642	0.000	0.000	No	No	No	No	1.98
B95	18.142	0.000	0.000	No	No	No	No	2.00
B98	9.800	0.000	0.000	No	No	No	No	2.00
B99	8.808	0.000	0.000	No	No	No	No	1.98

**Table 2c:** Summarized results of cell-free DNA and iChorCNA of CCA group (36 samples).

Sample ID	Cell-free DNA concentration (ng/ml plasma)	Circulating tumor DNA concentration (ng/ml plasma)	Tumor fraction (%)	SCNAs	Loss (Chromosome no.)	Gain (Chromosome no.)	Amplification (Chromosome no.)	Average tumour ploidy
C64	148.167	10.438	7.045	Yes	6,17,18,X	8	No	1.89
C65	44.800	0.000	0.000	Yes	No	19	No	2.01
C66	51.217	12.968	25.320	Yes	3,6,13,X	3,17	No	2.01
C67	76.417	13.426	17.570	Yes	1-5,7–14,16-18, 20,21	1-4,6–10,12-14, 16–20,22,X	5,19,20	1.95
C68	21.175	3.714	17.540	Yes	1-6,12,14,15,22	5,12,19	1,2,4,6	1.79
C69	8.750	0.000	0.000	Yes	6	No	No	2.00
C70	55.708	8.835	15.860	Yes	1,6,17,18,20	1-4,6–8,10-14, 16–18,20-22,X	1,19	2.65
C71	20.358	0.000	0.000	Yes	No	17,19	No	2.01
C72	39.783	2.739	6.885	Yes	6,10,13,14,18, 21,X	1,2,7,8,19	3,18	2.16
C73	54.075	0.000	0.000	No	No	No	No	2.00
C74	11.083	0.000	0.000	Yes	6	19	No	2.01
C75	99.750	19.631	19.680	Yes	1,4–10,14, 17-19,X	1,5,13	No	1.86
C76	63.000	0.000	0.000	Yes	6	19	No	2.00
C77	236.250	101.422	42.930	Yes	1-4,6,9–12,18, 20,21	1-6,8,9,12, 16-18,20,22,X	1,6,8,12,18,X	2.20
C78	24.267	2.754	11.350	Yes	1,3,5,7,9,14,17, 18,20	3,6,7,8,16,17,20	3,6,7	1.94
C79	101.500	0.000	0.000	Yes	No	19	No	2.00
C80	47.775	3.287	6.881	Yes	3-6,8–10,14,15, 21,22	3,7,8,20	5,8,19	1.89
C81	22.283	6.271	28.140	Yes	No	1,2,5,7,8,10,12, 13,15-18	4	1.78
C82	120.750	6.146	5.090	Yes	No	12,17,19,21	No	2.05
C83	13.533	0.000	0.000	Yes	No	19	No	2.01
C84	31.208	4.132	13.240	Yes	1-4,6,8–12,14, 17–20,22	2,3,7,8,12,14, 17,18,19	6, 8	1.87
C85	49.817	2.823	5.667	Yes	1-3,6,8,11,13, 14,16–18,20,X	1,7,17	No	1.67
C86	195.417	68.083	34.840	Yes	1,4–11,13-15, 17–22,X	6,7,10,11,17-19	4,6,8	1.79
C87	145.250	8.088	5.568	Yes	1,3,5,6,10,13, X	1,13,16,19, X	No	1.88
C88	254.917	17.411	6.830	Yes	6	1,4,5,12,13, 16-21, 22,X	No	2.11
C89	48.008	0.000	0.000	Yes	6	No	No	2.00
C90	8.633	0.000	0.000	Yes	No	19	No	2.03
C91	805.000	300.748	37.360	Yes	2-6, 8–13,16,17, 19,21,22,X	1,3,4,6–8,11, 13,15,16,20,21	1,12,16,19	2.04
C92	59.500	4.263	7.165	Yes	No	7,12	No	2.01
C93	23.508	4.586	19.510	Yes	3-10,13–19,21,X	2,6,17,19,20,22	3,5,8,10,12	1.80
C94	199.500	35.91	18.000	Yes	1,4–6,8,9,13, 14,16,17,20	1,2,5,11,12, 18-22, X	3,5–9,14,16–18, 20,X	2.58
C95	25.958	4.4	16.950	Yes	1-3,6,8,9,14, 15,20-22	1-3,7,9–13, 17,19,20,22,X	1,2,3,5,7,11,12, 19,20,	2.23
C96	56.058	6.077	10.840	Yes	1-6,8,9,11,13–19,21,X	1,3,4,6,711,12, 14,17,19	15,18	1.94
C97	133.583	7.004	5.243	Yes	6	1-4,6–13,15-17, 19–21,X	19	2.67
C98	77.000	9.101	11.820	Yes	1,6,9,14,17,X	1,2,4,8,9,12,15, 17-22	7	2.45
C99	44.158	0.000	0.000	Yes	6	19	No	2.02

**Table 3:** Descriptive statistics of cell-free DNA (cfDNA) and circulating tumor DNA (ctDNA) concentrations (ng/ml plasma), tumor fractions (%), and average ploidy numbers.

	cfDNA concentration			ctDNA concentration			% Tumor fraction			Average ploidy number		
	N	OV	CCA	N	OV	CCA	N	OV	CCA	N	OV	CCA
**No. of subject**	43	36	36	43	36	36	43	36	36	43	36	36
**Minimum**	2.917	0.000	8.633	0.000	0.000	0.000	0.000	0.000	0.000	1.99	1.97	1.67
**Maximum**	20.710	22.810	805.000	0.479	0.912	300.748	4.979	5.623	42.930	2.06	2.66	2.67
**Range**	17.790	22.810	796.400	0.479	0.912	300.748	4.979	5.623	42.930	0.07	0.69	1.00
**Median**	6.475	9.363	52.646	0.000	0.000	4.332	0.000	0.000	6.965	2.00	2.00	2.01
**Mean/Avg**	7.879	9.826	94.950	0.049	0.051	18.452	0.693	0.501	11.040	2.005	2.025	2.036
**SD.**	4.019	5.482	138.000	0.125	0.186	52.389	1.598	1.453	11.550	0.016	0.110	0.231
**SE.**	0.6130	0.9136	23.0000	0.019	0.031	8.732	0.244	0.242	1.925	0.003	0.018	0.039

**Table 4:** Pairwise comparison of health status with plasma ctDNA, tumor fraction of plasma cfDNA, and difference in values of ploidy number^∗^.

Health status	N	Mean ± SD (mean rank)		
		Plasma ctDNA	Tumor fraction of plasma cfDNA	Difference in values of ploidy number
N	43	0.049 ± 0.124 (47.65)^a^	0.692 ± 1.597 (47.30)^a^	0.009 ± 0.014 (42.36)^a^
OV	36	0.051 ± 0.186 (44.46)^a^	0.500 ± 1.453 (45.11)^a^	0.030 ± 0.108 (52.92)^a^
CCA	36	18.451 ± 52.389 (83.90)^b^	11.036 ± 11.548 (83.67)^b^	0.144 ± 0.182 (81.76)^b^

^∗^Kruskal-Wallis test followed by a Dunn’ s pairwise comparison was used to find statistically significant differences between health status groups.

^a,b^ Indicate significant differences (*P* < 0.05).

**Table 5:** Summary of diagnostic parameters of plasma cell-free DNA (cfDNA) concentration, circulating tumor DNA (ctDNA) concentration, tumor fraction, and average ploidy number.

Diagnostic parameter/Group	N vs CCA	N vs CCA	OV vs CCA	OV vs CCA	N & OV vs CCA	N & OV vs CCA	N vs CCA	N vs CCA	OV vs CCA	OV vs CCA	N & OV vs CCA	N & OV vs CCA
	cfDNA						ctDNA					
***Cut-off value***	19.78	20.94	20.21	23.16	20.94	23.16	0.479	1.609	0.765	1.826	0.765	1.826
***Number of true positive***	32	31	32	29	31	29	25	25	25	25	25	25
***Number of true negative***	42	43	35	36	78	79	43	43	35	36	78	79
***Number of false positive***	1	0	1	0	1	0	0	0	1	0	1	0
***Number of false negative***	4	5	4	7	5	7	11	11	11	11	11	11
***% Sensitivity***	88.89	86.11	88.89	80.56	86.11	80.56	69.44	69.44	69.44	69.44	69.44	69.44
***% Specificity***	97.67	100.00	97.22	100.00	98.73	100.00	100.00	100.00	97.22	100.00	98.73	100.00
***Positive Likelihood Ratio***	38.22	N/A	32.00	N/A	68.03	N/A	N/A	N/A	25	N/A	54.86	N/A
***Negative Likelihood Ratio***	0.11	0.14	0.11	0.19	0.14	0.19	0.31	0.31	0.31	0.31	0.31	0.31
***% Positive Predictive Value***	96.97	100	96.97	100	96.88	100	100.00	100.00	96.15	100.00	96.15	100.00
***% Negative Predictive Value***	91.30	89.58	89.74	83.72	93.98	91.86	79.63	79.63	76.09	76.60	87.64	87.78
***% Accuracy***	93.67	93.67	93.06	90.28	94.78	93.91	86.08	86.08	83.33	84.72	89.57	90.43
	***Tumor fraction***						***Ploidy number***					

***Cut-off value***	4.724	5.035	4.746	5.645	4.746	5.645	>2.10 & <1.90		>2.10 & <1.90		>2.10 & <1.90	
***Number of true positive***	25	25	25	22	25	22		18		18		18
***Number of true negative***	42	43	35	36	77	79		43		35		78
***Number of false positive***	1	0	1	0	2	0		0		1		1
***Number of false negative***	11	11	11	14	11	14		18		18		18
***% Sensitivity***	69.44	69.44	69.44	61.11	69.44	61.11		50.00		50.00		50.00
***% Specificity***	97.67	100.00	97.22	100.00	97.47	100.00		100.00		97.22		98.73
***Positive Likelihood Ratio***	29.86	N/A	25	N/A	27.43	N/A		N/A		18.00		39.50
***Negative Likelihood Ratio***	0.31	0.31	0.31	0.39	0.31	0.39		0.50		0.51		0.51
***% Positive Predictive Value***	96.15	100.00	96.15	100.00	92.59	100.00		100.00		94.74		94.74
***% Negative Predictive Value***	79.25	79.63	76.09	72.00	87.50	84.95		70.49		66.04		81.25
***% Accuracy***	84.81	86.08	83.33	80.56	88.70	87.83		77.22		73.61		83.48

N/A = Not applicable.

### 3.3 Increment of plasma ctDNA concentration in CCA

The ctDNA concentration (Fig. 1C), the averages and ranges (max–min ng/ml) of ctDNA concentrations were highest in the CCA group (average = 18.450, range = 0.000–300.748) when compared to the N group (average = 0.049, range = 0.000–0.479) and the OV group (average = 0.051, range = 0.000–0.912) (Table 3). A Kruskal-Wallis test provided strong evidence of a difference in the mean ranks between the health status, which meant at least two groups differed (H [2] = 47.95, *P* < 0.001) (Table 4). Dunn's pairwise comparisons were done for the three pairs of groups. Strong evidence (*P* < 0.001, adjusted by the Bonferroni correction) of differences between the N and CCA groups and between the OV and CCA groups was found. The mean ranks for the N and OV groups were 47.65 and 44.46, respectively, while the mean rank of the CCA group was 83.90. There was no evidence of any differences between the N and OV groups (*P* > 0.05). The diagnostic parameters are summarized in Table 5. In a differential diagnosis of the CCA group from the OV and N groups, 69.44% sensitivity, 100% specificity, 100% PPV, 87.78% NPV, and 90.43% accuracy were obtained by using 1.826 ng/ml plasma as a cut-off. The AUC of the ROC curve of the CCA group vs the N group was 0.8224 (Fig. 3) while that of the CCA group vs the OV group was 0.8345 (Fig. 3). To differential diagnose the CCA group from the N and OV groups, the AUC of the ROC curves was 0.8279 (Fig. 3). The level of plasma ctDNA concentration showed promising results in the differential diagnosis of the CCA group, like the plasma cfDNA concentration. However, the AUC of the ROC curve showed that the differentiation between the N and OV groups was limited (0.5349) (Fig. 3).

### 3.4 Tumor fraction of plasma cfDNA

Tumor fraction was detected in 6 (13.95%), 4 (11.11%), and 25 (69.44%) subjects in the N, OV, and CCA groups, respectively. The averages and ranges (min-max) of the percentage of tumor fraction of plasma cfDNA in the N, OV, and CCA groups were 0.693 (0.000–4.979), 0.501 (0.000–5.623), and 11.040 (0.000–42.930), respectively (Table 3). The result indicated that the tumor fraction percentage of the plasma cfDNA was highest in the CCA group (Fig. 1B). Strong evidence of differences (H [2] = 46.07, *P* < 0.001) between the mean ranks of at least one pair of groups was observed. Dunn's pairwise tests provided strong evidence (*P* < 0.001, adjusted by the Bonferroni correction) of differences between the N and CCA groups and between the OV and CCA groups. There was no evidence of any differences between the N and OV groups (*P* < 0.05). The mean ranks for the N, OV, and CCA groups were 47.30, 45.11, and 83.67, respectively. In a differential diagnosis of the CCA group from the OV and N groups, 61.11% sensitivity, 100% specificity, 100% PPV, 84.95% NPV, and 87.83% accuracy were obtained (Table 5). The AUC of the ROC curve indicated the accepted accuracy of differential diagnosis of the CCA group from the N and OV groups (Fig. 3) but not for the N group vs the OV group (Fig. 3). The highest AUC of the ROC curve (0.8279) was observed in both the OV group vs the CCA group and the N and OV groups vs the CCA group (Fig. 3).

### 3.5 Ploidy number

The averages and the ranges (maximum-minimum) of the ploidy number were 2.005 (1.97–2.66), 2.025 (1.97–2.66), and 2.036 (1.67–2.67) in the N, OV, and CCA groups, respectively (Fig. 1D). The range of the N and OV groups were 0.07 and 0.69, respectively, while the highest range of 1.00 was observed in the CCA group (Table 3). Using the change in the average ploidy number of >2.10 and < 1.90 as a cut-off, 18 of the 36 control subjects (50%) in the CCA group were detected with a single false-positive case in the OV group (Table 5). Using this cut-off, all the subjects in the N group were true negative subjects. In terms of the association between the health status and the difference in values of the ploidy number, strong evidence suggested that at least two of the groups differed (H [2] = 30.29, *P* < 0.001). The differences in the values of the ploidy number in the CCA group were found to be markedly different from that in the N and OV groups. The mean ranks for the N, OV, and CCA groups were found to be 42.36, 52.92, and 81.76, respectively. In a differential diagnosis of the CCA group from the OV and N groups, 50% sensitivity, 98.73% specificity, 94.74% PPV, 81.25% NPV, and 83.48% accuracy were obtained (Table 5). From the ploidy number, the high specificity of CCA detection was obtained by using the average ploidy number with limited sensitivity.

### 3.6 Somatic copy number alteration (SCNA)

The iChorCNA detected SCNA (loss, gain, and amplification) for 0.0% (0/43) (Table 2A), 8.3% (3/36) (Table 2B), and 97.2% (35/36) (Table 2C) in the N, OV, and CCA groups, respectively (S1-S3 Figures). No SCNA was found in the N group (Table 2A). In the OV group, a single loss-case and three gain-cases were observed at chromosome 5 (Table 2B). In the CCA group, SCNAs were found in several chromosomes (Table 2C and S4). A loss of the genome of CCA was detected in 27 of the 36 (75.0%) subjects. A gain of the genome of CCA was detected in 33 of the 36 (91.7%) subjects. An amplification of the genome of CCA was detected in 17 of the 36 (47.2%) subjects. The gain of the genome was detected in half of the CCA subjects (50.0%). The gain of chromosomes 16, 18, 20, and X was found only in the CCA group. There was no specific pattern of SCNA in the CCA group. It is possible that SCNAs were detected in all the chromosomes. However, at least 55% of the subjects in the CCA group experienced a loss of short arm 6p21.31–6p22.1. For 6p22.1, 23 of the 36 (63.9%) subjects were lost in the genome. For 6p21.31, 21 of 36 (58.3%) subjects were lost in the genome. The gain of chromosome 19 was most frequently found in in the CCA group (22 of the 36 (61.1%) subjects). For amplification, the most frequently found chromosomes were chromosomes 6, 8, and 19, all of them in 6 of the 36 (16.7%) subjects in the CCA group. In terms of the association between health status and SCNA, a Pearson's chi-square test revealed strong evidence indicating the association was present (*P* < 0.001). The odds ratios for the association between health status and SCNA are presented in Table 6. Compared to the N group, the CCA group was found to be statistically significant, with an odds ratio of 11.688 (*P* < 0.001, 95% CI: 3.984, 34.290). However, the OV group was found not to be statistically significant, with an odds ratio of 0.643 (*P* = 0.511, 95% CI: 0.172, 2.401).

**Table 6:** Association between health status and SCNA.^a^

Characteristic	N	SCNA							
		Yes		No		Chi-square (*P*-value)	OR	95% CI	
		N	%	N	%			lower	upper
Health status						<0.001			
Non (Ref.)	43	7	16.3	36	83.7		*t*_wald_ = 29.502, df = 2, *P* < 0.001	–	–
OV	36	4	11.1	32	88.9		0.643	0.172	2.401
CCA	36	25	69.4	11	30.6		11.688^b^	3.984	34.290
Total	115	36	31.3	79	68.7				

a Association results obtained from Pearson's chi-square and binary logistic regression analyses.

b Indicate high significant differences (*P* < 0.001).

## 4 Discussion

Several attempts have been made to develop an effective diagnosis for CCA. Several biomarkers have been identified and investigated for their sensitivity, specificity, and accuracy, but they are still being developed [[10], [11], [12]]. Recently, the levels and SCNA of plasma cfDNA or tissue of cholangiocarcinoma have been intensively investigated [13,14]. Despite the extensive use of cell-free DNA in liquid biopsy, a non-invasive testing method, its application for cancer diagnosis is not well-established for routine use. This limitation may be attributed to the high cost and time required for deep DNA sequencing, which poses a major obstacle to patient access to the test, as well as targeted therapy, especially in developing countries. Furthermore, the targeted therapy is not available for CCA at present. Hence, this study attempted to utilize ULP-WGS, a method at least 5 times cheaper than deep sequencing exome or WGS, which allows patient access or support by the national health policy. In our study, both cfDNA and ctDNA concentrations were increased in the plasma of CCA patients, with high sensitivity, high specificity, and high accuracy. This increment was consistent with other cancers [[15], [16], [17]]. The increase in plasma cfDNA and ctDNA in CCA patients may be due to the rate of cell death being higher than normal, which releases the cfDNA into the bloodstream [6,[18], [19], [20]]. Although the increase in cell-free DNA (cfDNA) was significantly higher in the CCA group, a slight increase was also observed in some healthy subjects and *O. viverrini*-infected individuals. Besides cancer, elevated cfDNA levels can occur in other diseases or conditions, such as autoimmune disorders, infections, sepsis, trauma, surgery, cardiovascular diseases, transplant rejection, and chronic inflammatory diseases [[21], [22], [23]]. The *O. viverrini*-infected subjects were asymptomatic, which might have allowed chronic infection in some cases, potentially stimulating inflammation, leading to cell lysis, and releasing cfDNA. It is also possible that some subjects were in the early stages of CCA, which ultrasonography could not yet detect. However, underlying diseases or some condition may possible account for this increment. In the healthy control group, the exact reason for the increase in cfDNA was unclear. The factors such as exercise, aging, and stress might also contribute to the elevated levels of cfDNA [24,25]. Measurement of the plasma cfDNA is simple, inexpensive, and quick method that can be done with a ready-to-use kit and requires only an absorbance reader. The cost of measuring plasma cfDNA concentration is less than ten dollars, making it suitable for screening suspected cancer patients or an annual health check-up. Nonetheless, the use of cfDNA for the CCA diagnostic approach, the baseline level, and the kinetic and biological variations such as age, gender, race, and diseases are not well established and needs to be investigated further. Recently, Adalsteinsson et al. developed a novel powerful method, ULP-WGS, which is analyzed by the iChorCNA software [7]. This time-saving and cost-effectiveness technique was applied for the detection of tumor fraction, ploidy number, and SCNA (loss, gain, and amplification). The ULP-WGS and iChorCNA software could distinguish the CCA group from the N and OV groups with high specificity based on the % tumor fraction, calculated ctDNA, ploidy change, and SCNA of the plasma cfDNA. The CCA group showed SCNA losses and gains in most subjects, especially in chromosomes 16, 18, 20, and X, which were unique to this group. We also observed a loss of chromosome 6 short arm with the HLA genes, which could impair the immune response to cancer. Subjects in the CCA group experienced a loss of short arm 6p21.31–6p22.1 (chromosome 6 sequence of 28–34 MB) (ftp.ncbi.nlm.nih.gov/pub/gdp/ideogram_9606_GCF_000001305.15_850_V1, retrieved May 16, 2022). This region included *HLA-A*, *HLA-B*, *HLA-C*, *HLA-E*, *HLA-F*, *HLA-G*, *HLA-DR*, and *HLA-DQ* genes [26]. For 6p22.1 is the location of the *HLA-A*, *HLA-E*, *HLA-F*, and *HLA-G* genes while 6p21.31 is the location of the *HLA-DO*, *HLA-DP*, *HLA-DQ*, and *HLA-DR* genes. We achieved a highly sensitive, specific, and accurate diagnosis of CCA using cfDNA and loss of chromosome 6. These parameters could distinguish the CCA group from the N and OV groups. Therefore, we suggest that cfDNA concentration can be used for screening tests, while ULP-WGS with iChorCNA can be used for confirmation of CCA. The SCNA losses and gains especially chromosome 6 could serve as potential biomarkers for CCA diagnosis. Our study demonstrated that combining cfDNA concentration and ULP-WGS can diagnose CCA and differentiate opisthorchiasis viverrini with a non-invasive, high-performance, time-saving, and cost-effective approach. Regarding this complexity and heterogeneity of cancer, the application of Artificial Intelligence (AI) in predicting genetic diseases, including genetically altered conditions like cancer, holds significant potential. AI can play a crucial role in both diagnosis and treatment, particularly in the selection of targeted therapies based on specific genetic alterations [27,28].

## 5 Conclusion

The diagnosis of CCA is crucial and urgently needed to prevent the development of CCA. However, CCA diagnosis is not readily available, possibly due to issues of efficacy, cost, and testing duration. Recently, the use of cfDNA can detect early cancer. Therefore, this study aims to investigate the potential biomarker of cfDNA in both quantitative and qualitative manners.

The application of plasma cfDNA concentration by fluorometry can differentiate CCA from OV and N effectively. The application of ULP-WGS with iChorCNA for analyzing % tumor fraction, calculating ctDNA concentration, tumor ploidy, and SCNA were also effective for the differential diagnosis of CCA from OV and N. Loss, gain, and amplification of chromosomes were detected in the CCA. The loss of the short arm of chromosome 6 was particularly pronounced and suggested a specific pattern for CCA.

The application of plasma cfDNA concentration fluorometry and ULP-WGS with iChorCNA is a potential biomarker for CCA with efficacy, time-saving, and cost-effectiveness.

## CRediT authorship contribution statement

**Sattrachai Prasopdee:** Writing – review & editing, Validation, Software, Methodology, Investigation, Funding acquisition, Formal analysis, Data curation, Conceptualization. **Sissades Tongsima:** Writing – original draft, Supervision, Methodology, Conceptualization. **Montinee Pholhelm:** Methodology, Investigation, Formal analysis, Data curation. **Siraphatsorn Yusuk:** Methodology, Investigation, Formal analysis, Data curation. **Sithichoke Tangphatsornruang:** Software, Methodology, Formal analysis, Data curation. **Kritiya Butthongkomvong:** Methodology, Investigation, Formal analysis, Data curation. **Teva Phanaksri:** Methodology, Investigation, Data curation. **Anthicha Kunjantarachot:** Methodology, Investigation, Data curation. **Jutharat Kulsantiwong:** Methodology, Investigation, Data curation. **Smarn Tesana:** Supervision, Conceptualization. **Thanakrit Sathavornmanee:** Methodology, Investigation, Data curation. **Veerachai Thitapakorn:** Writing – review & editing, Validation, Supervision, Software, Methodology, Investigation, Funding acquisition, Formal analysis, Data curation.

## Patient consent for publication

Not applicable.

## Availability of data and materials

All data generated or analyzed in this study are all included in this publication and supplementary files.

## Ethics approval and consent to participate

This study was reviewed and approved by Human Ethics Committee of Udonthani Cancer Hospital, Udon Thani, Ministry of Public Health, Thailand with the approval number: (UCH-CT 11/2563), dated May 29, 2020. All participants provided written informed consent to participate in the study and for their data to be reported as a summary, not specific individuals.

## Funding

1. Chulabhorn International College of Medicine, 10.13039/501100005790Thammasat University, Fund Contract No. T3/2562 to Dr. Veerachai Thitapakorn
2. Thammasat Research Unit in Opisthorchiasis, Cholangiocarcinoma, and Neglected Parasitic Diseases, 10.13039/501100005790Thammasat University (TRU-OCN) to Dr. Veerachai Thitapakorn and Dr. Sattrachai Prasopdee
3. Thai Government Research Fund through 10.13039/501100005790Thammasat University, Fund Contract No. 36/2562 to Dr. Sattrachai Prasopdee

## Declaration of competing interest

The authors declare the following financial interests/personal relationships which may be considered as potential competing interests:Veerachai Thitapakorn reports financial support was provided by 10.13039/501100005790Thammasat University. Veerachai Thitapakorn reports a relationship with Thammasat University that includes: employment. The author declares, there is no conflict of interest for this study. If there are other authors, they declare that they have no known competing financial interests or personal relationships that could have appeared to influence the work reported in this paper.
