# Supplementary figures and images for: Biomarker potential of plasma cell-free DNA for cholangiocarcinoma
Source: Heliyon. 2024 Dec 6;10(24):e41008. doi: 10.1016/j.heliyon.2024.e41008 (PMC11681853; doi:10.1016/j.heliyon.2024.e41008)

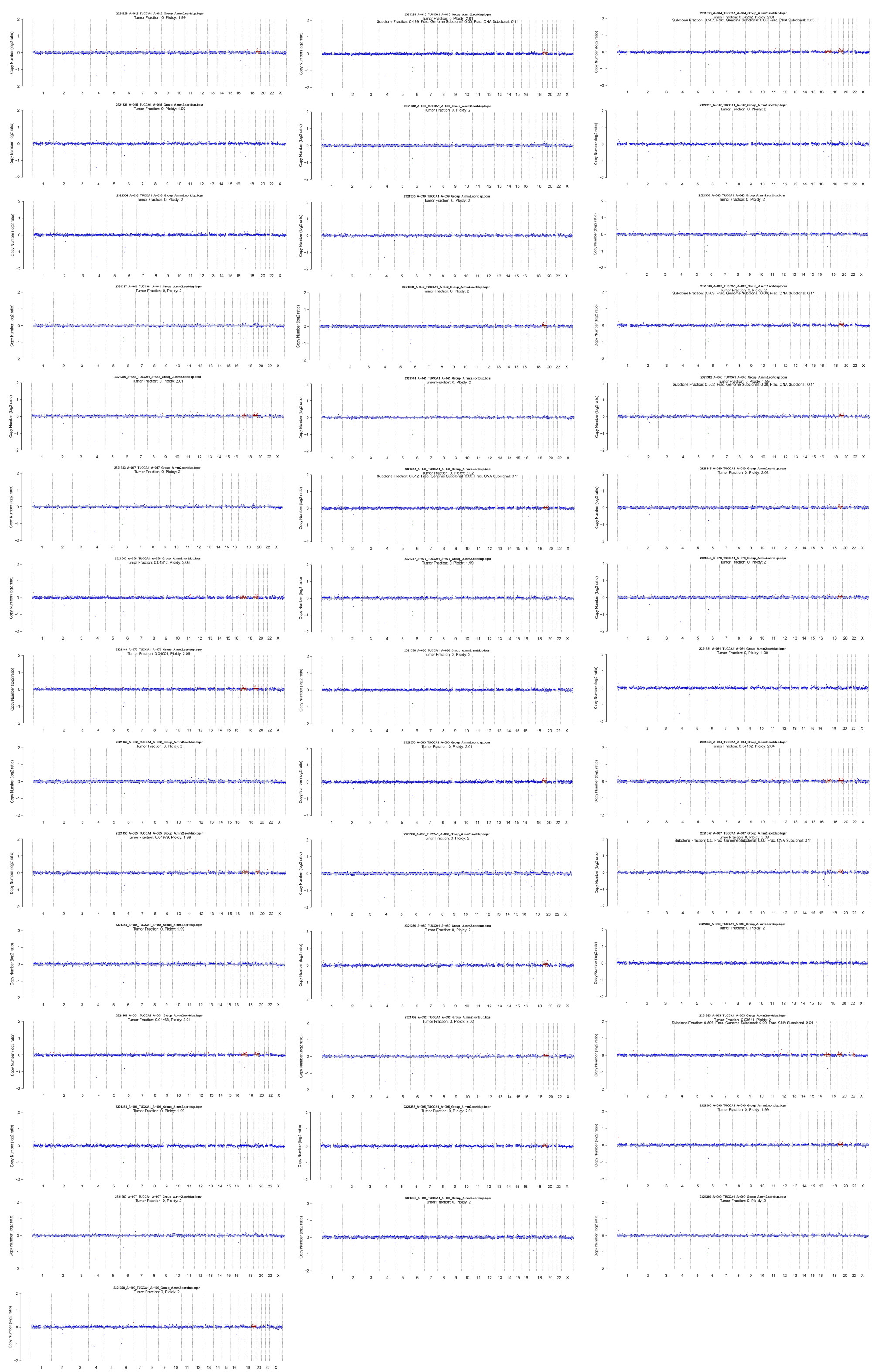

Supplement: Multimedia component 1 [file mmc1.pdf]

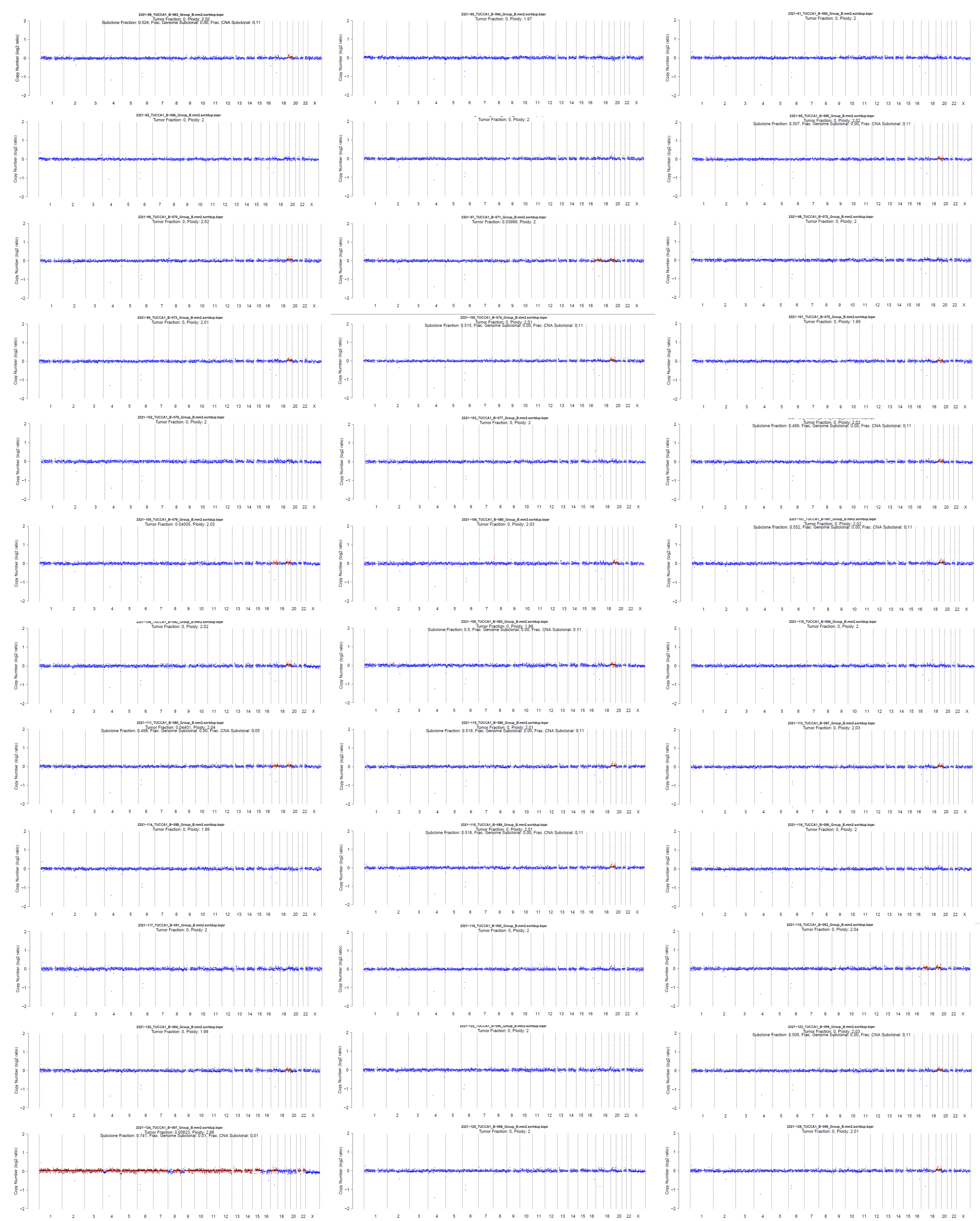

Supplement: Multimedia component 2 [file mmc2.pdf]

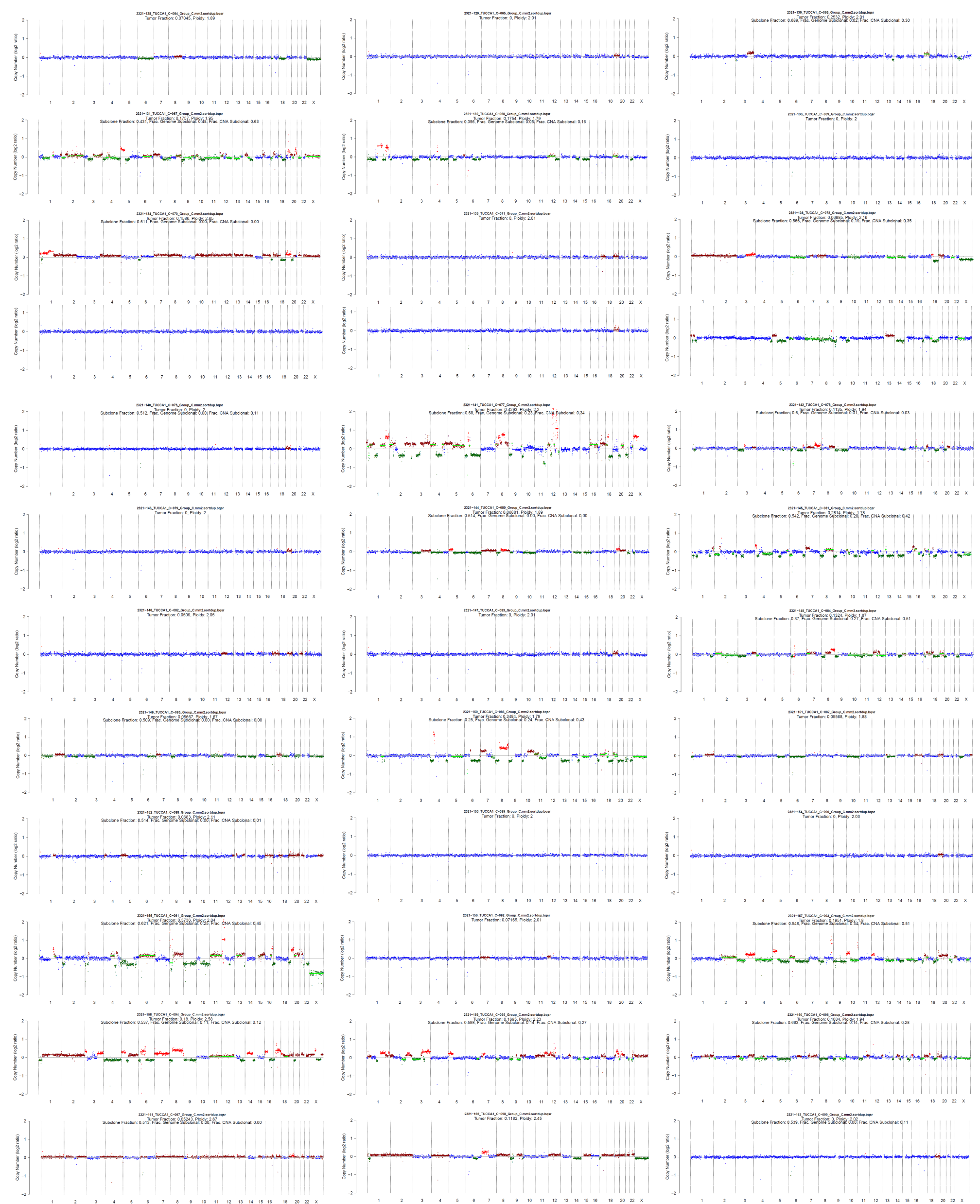

Supplement: Multimedia component 3 [file mmc3.pdf]
