# Supplementary material for: Biomarker potential of plasma cell-free DNA for cholangiocarcinoma
Source: Heliyon. 2024 Dec 6;10(24):e41008. doi: 10.1016/j.heliyon.2024.e41008 (PMC11681853; doi:10.1016/j.heliyon.2024.e41008)
Supplement: Multimedia component 4 [file mmc4.docx]

S4 Somatic copy number alteration (SCNA) Raw data

| ID | chromosome number | start | end | num.mark | seg.median.logR | copy.number | call | subclone.status | logR_Copy_Number | Corrected_Copy_Number | Corrected_Call |
| --- | --- | --- | --- | --- | --- | --- | --- | --- | --- | --- | --- |
| B71 | 17 | 1,000,001 | 81,000,000 | 80 | 0.026440272 | 3 | GAIN | FALSE | 2.9245848 | 3 | GAIN |
| B71 | 19 | 1,000,001 | 59,000,000 | 58 | 0.036076032 | 3 | GAIN | FALSE | 3.2667603 | 3 | GAIN |
| B79 | 17 | 1,000,001 | 81,000,000 | 80 | 0.011442014 | 3 | GAIN | FALSE | 2.4258111 | 3 | GAIN |
| B79 | 19 | 1,000,001 | 59,000,000 | 58 | 0.037902484 | 3 | GAIN | FALSE | 3.3580472 | 3 | GAIN |
| B97 | 1 | 1,000,001 | 248,000,000 | 247 | 0.019893322 | 3 | GAIN | FALSE | 3.1648287 | 3 | GAIN |
| B97 | 2 | 1,000,001 | 243,000,000 | 242 | 0.008866804 | 3 | GAIN | FALSE | 2.8851547 | 3 | GAIN |
| B97 | 3 | 1,000,001 | 197,000,000 | 196 | 0.008056009 | 3 | GAIN | FALSE | 2.8646742 | 3 | GAIN |
| B97 | 4 | 35,000,001 | 191,000,000 | 156 | 0.009152457 | 3 | GAIN | FALSE | 2.892373 | 3 | GAIN |
| B97 | 5 | 18,000,001 | 180,000,000 | 162 | 0.003892391 | 3 | GAIN | FALSE | 2.7596829 | 3 | GAIN |
| B97 | 5 | 1,000,001 | 17,000,000 | 16 | -0.135249775 | 1 | HETD | TRUE | 0.015625 | 1 | HETD |
| B97 | 6 | 1,000,001 | 170,000,000 | 169 | 0.020460301 | 3 | GAIN | FALSE | 3.1792673 | 3 | GAIN |
| B97 | 7 | 1,000,001 | 159,000,000 | 158 | 0.009045698 | 3 | GAIN | FALSE | 2.8896751 | 3 | GAIN |
| B97 | 8 | 59,000,001 | 145,000,000 | 86 | 0.013597401 | 3 | GAIN | FALSE | 3.0048787 | 3 | GAIN |
| B97 | 9 | 33,000,001 | 141,000,000 | 108 | 0.01974761 | 3 | GAIN | FALSE | 3.161119 | 3 | GAIN |
| B97 | 10 | 1,000,001 | 135,000,000 | 134 | 0.016563582 | 3 | GAIN | FALSE | 3.0801486 | 3 | GAIN |
| B97 | 11 | 2,000,001 | 134,000,000 | 132 | -0.004092926 | 3 | GAIN | FALSE | 2.5591681 | 3 | GAIN |
| B97 | 12 | 1,000,001 | 132,000,000 | 131 | 0.011811344 | 3 | GAIN | FALSE | 2.9596303 | 3 | GAIN |
| B97 | 13 | 20,000,001 | 114,000,000 | 94 | 0.00537981 | 3 | GAIN | FALSE | 2.7971554 | 3 | GAIN |
| B97 | 14 | 21,000,001 | 106,000,000 | 85 | 0.011734881 | 3 | GAIN | FALSE | 2.9576944 | 3 | GAIN |
| B97 | 15 | 25,000,001 | 102,000,000 | 77 | 0.033945172 | 3 | GAIN | FALSE | 3.5243483 | 3 | GAIN |
| B97 | 17 | 1,000,001 | 81,000,000 | 80 | 0.027554033 | 3 | GAIN | FALSE | 3.3603954 | 3 | GAIN |
| B97 | 21 | 16,000,001 | 48,000,000 | 32 | 0.007369286 | 3 | GAIN | FALSE | 2.8473366 | 3 | GAIN |
| B97 | 22 | 22,000,001 | 50,000,000 | 28 | 0.036469888 | 3 | GAIN | FALSE | 3.5893156 | 3 | GAIN |
| C64 | 6 | 1,000,001 | 170,000,000 | 169 | -0.044478288 | 1 | HETD | FALSE | 1.0348421 | 1 | HETD |
| C64 | 8 | 65,000,001 | 145,000,000 | 80 | 0.046225392 | 3 | GAIN | FALSE | 2.8143999 | 3 | GAIN |
| C64 | 17 | 1,000,001 | 18,000,000 | 17 | -0.05093002 | 1 | HETD | FALSE | 0.9124737 | 1 | HETD |
| C64 | 18 | 1,000,001 | 78,000,000 | 77 | -0.0572144 | 1 | HETD | FALSE | 0.7938045 | 1 | HETD |
| C64 | X | 4,000,001 | 151,000,000 | 147 | -0.094089685 | 1 | HETD | FALSE | 0.1078009 | 1 | HETD |
| C65 | 19 | 1,000,001 | 59,000,000 | 58 | 0.04593541 | 3 | GAIN | FALSE | 3.0583066 | 3 | GAIN |
| C66 | 3 | 126,000,001 | 197,000,000 | 71 | 0.174387206 | 3 | GAIN | FALSE | 3.0313435 | 3 | GAIN |
| C66 | 3 | 1,000,001 | 10,000,000 | 9 | -0.20383694 | 1 | HETD | FALSE | 0.9720606 | 1 | HETD |
| C66 | 6 | 28,000,001 | 34,000,000 | 6 | -3.95720141 | 1 | HETD | TRUE | 0.015625 | 1 | HETD |
| C66 | 13 | 96,000,001 | 114,000,000 | 18 | -0.174431929 | 1 | HETD | FALSE | 1.1135259 | 1 | HETD |
| C66 | 17 | 26,000,001 | 81,000,000 | 55 | 0.119234417 | 3 | GAIN | TRUE | 3.0113727 | 3 | GAIN |
| C66 | X | 4,000,001 | 46,000,000 | 42 | -0.106535815 | 1 | HETD | FALSE | 1.4513974 | 1 | HETD |
| C67 | 1 | 150,000,001 | 163,000,000 | 13 | 0.081711362 | 3 | GAIN | TRUE | 3.4194061 | 3 | GAIN |
| C67 | 1 | 234,000,001 | 248,000,000 | 14 | 0.146189731 | 3 | GAIN | FALSE | 3.1595328 | 3 | GAIN |
| C67 | 1 | 43,000,001 | 120,000,000 | 77 | -0.051890855 | 1 | HETD | TRUE | 0.9568054 | 1 | HETD |
| C67 | 2 | 39,000,001 | 65,000,000 | 26 | 0.145399134 | 3 | GAIN | FALSE | 3.1526623 | 3 | GAIN |
| C67 | 2 | 65,000,001 | 138,000,000 | 73 | 0.064880881 | 3 | GAIN | TRUE | 3.0964823 | 3 | GAIN |
| C67 | 2 | 138,000,001 | 161,000,000 | 23 | 0.127978592 | 3 | GAIN | FALSE | 3.0022246 | 3 | GAIN |
| C67 | 2 | 161,000,001 | 233,000,000 | 72 | 0.072469457 | 3 | GAIN | TRUE | 3.2416169 | 3 | GAIN |
| C67 | 2 | 1,000,001 | 39,000,000 | 38 | -0.029953234 | 1 | HETD | TRUE | 1.3456819 | 1 | HETD |
| C67 | 3 | 94,000,001 | 103,000,000 | 9 | 0.131266494 | 3 | GAIN | FALSE | 3.0304789 | 3 | GAIN |
| C67 | 3 | 103,000,001 | 176,000,000 | 73 | 0.06762633 | 3 | GAIN | TRUE | 3.148902 | 3 | GAIN |
| C67 | 3 | 176,000,001 | 197,000,000 | 21 | 0.148248457 | 3 | GAIN | FALSE | 3.1774414 | 3 | GAIN |
| C67 | 3 | 38,000,001 | 90,000,000 | 52 | -0.101787906 | 1 | HETD | FALSE | 1.1790319 | 1 | HETD |
| C67 | 4 | 62,000,001 | 80,000,000 | 18 | 0.069113749 | 3 | GAIN | TRUE | 3.1773434 | 3 | GAIN |
| C67 | 4 | 2,000,001 | 31,000,000 | 29 | -0.114124391 | 1 | HETD | FALSE | 1.0891153 | 1 | HETD |
| C67 | 4 | 33,000,001 | 62,000,000 | 29 | -0.024276229 | 1 | HETD | TRUE | 1.447282 | 1 | HETD |
| C67 | 4 | 80,000,001 | 156,000,000 | 76 | -0.045843078 | 1 | HETD | TRUE | 1.0634214 | 1 | HETD |
| C67 | 4 | 156,000,001 | 191,000,000 | 35 | -0.099547401 | 1 | HETD | FALSE | 1.1954449 | 1 | HETD |
| C67 | 5 | 50,000,001 | 99,000,000 | 49 | -0.129906032 | 1 | HETD | FALSE | 0.9752035 | 1 | HETD |
| C67 | 5 | 1,000,001 | 46,000,000 | 45 | 0.39725185 | 5 | HLAMP | FALSE | 5.5433343 | 6 | HLAMP |
| C67 | 6 | 56,000,001 | 155,000,000 | 99 | 0.042464286 | 3 | GAIN | TRUE | 2.6721874 | 3 | GAIN |
| C67 | 7 | 1,000,001 | 74,000,000 | 73 | 0.136682371 | 3 | GAIN | FALSE | 3.0771604 | 3 | GAIN |
| C67 | 7 | 98,000,001 | 159,000,000 | 61 | -0.110380598 | 1 | HETD | FALSE | 1.1163214 | 1 | HETD |
| C67 | 8 | 30,000,001 | 34,000,000 | 4 | 0.082064822 | 3 | GAIN | TRUE | 3.4262284 | 3 | GAIN |
| C67 | 8 | 84,000,001 | 145,000,000 | 61 | 0.065061218 | 3 | GAIN | TRUE | 3.0999225 | 3 | GAIN |
| C67 | 8 | 1,000,001 | 30,000,000 | 29 | -0.063224667 | 1 | HETD | TRUE | 0.7582018 | 1 | HETD |
| C67 | 9 | 105,000,001 | 132,000,000 | 27 | 0.046436025 | 3 | GAIN | TRUE | 2.7468836 | 3 | GAIN |
| C67 | 9 | 1,000,001 | 33,000,000 | 32 | -0.138572301 | 1 | HETD | FALSE | 0.9131783 | 1 | HETD |
| C67 | 9 | 33,000,001 | 105,000,000 | 72 | -0.036543465 | 1 | HETD | TRUE | 1.2282384 | 1 | HETD |
| C67 | 10 | 8,000,001 | 44,000,000 | 36 | 0.066658282 | 3 | GAIN | TRUE | 3.1304074 | 3 | GAIN |
| C67 | 10 | 1,000,001 | 8,000,000 | 7 | -0.027983956 | 1 | HETD | TRUE | 1.3808804 | 1 | HETD |
| C67 | 10 | 44,000,001 | 111,000,000 | 67 | -0.119043981 | 1 | HETD | FALSE | 1.053472 | 1 | HETD |
| C67 | 10 | 111,000,001 | 135,000,000 | 24 | -0.021380741 | 1 | HETD | TRUE | 1.499256 | 1 | HETD |
| C67 | 11 | 2,000,001 | 134,000,000 | 132 | -0.038903714 | 1 | HETD | TRUE | 1.1863071 | 1 | HETD |
| C67 | 12 | 1,000,001 | 6,000,000 | 5 | 0.227892437 | 3 | GAIN | FALSE | 3.8902417 | 3 | GAIN |
| C67 | 12 | 10,000,001 | 39,000,000 | 29 | 0.066848717 | 3 | GAIN | TRUE | 3.1340447 | 3 | GAIN |
| C67 | 12 | 67,000,001 | 72,000,000 | 5 | 0.1411302 | 3 | GAIN | FALSE | 3.1156291 | 3 | GAIN |
| C67 | 12 | 72,000,001 | 132,000,000 | 60 | -0.039625062 | 1 | HETD | TRUE | 1.1735056 | 1 | HETD |
| C67 | 13 | 81,000,001 | 97,000,000 | 16 | 0.051530181 | 3 | GAIN | TRUE | 2.8429905 | 3 | GAIN |
| C67 | 13 | 20,000,001 | 81,000,000 | 61 | -0.079165005 | 1 | HETD | TRUE | 0.4815046 | 1 | HETD |
| C67 | 14 | 21,000,001 | 51,000,000 | 30 | 0.114091209 | 3 | GAIN | FALSE | 2.8835926 | 3 | GAIN |
| C67 | 14 | 51,000,001 | 75,000,000 | 24 | -0.027416977 | 1 | HETD | TRUE | 1.3910233 | 1 | HETD |
| C67 | 14 | 75,000,001 | 106,000,000 | 31 | -0.145462613 | 1 | HETD | FALSE | 0.8641291 | 1 | HETD |
| C67 | 16 | 1,000,001 | 21,000,000 | 20 | 0.151506062 | 3 | GAIN | FALSE | 3.2058311 | 3 | GAIN |
| C67 | 16 | 85,000,001 | 90,000,000 | 5 | -0.103164237 | 1 | HETD | FALSE | 1.1689621 | 1 | HETD |
| C67 | 17 | 26,000,001 | 47,000,000 | 21 | 0.164848106 | 3 | GAIN | FALSE | 3.322777 | 3 | GAIN |
| C67 | 17 | 1,000,001 | 18,000,000 | 17 | -0.055444213 | 1 | HETD | TRUE | 0.8943716 | 1 | HETD |
| C67 | 18 | 1,000,001 | 25,000,000 | 24 | 0.045038054 | 3 | GAIN | TRUE | 2.7205686 | 3 | GAIN |
| C67 | 18 | 42,000,001 | 78,000,000 | 36 | -0.136124048 | 1 | HETD | FALSE | 0.9306629 | 1 | HETD |
| C67 | 19 | 22,000,001 | 43,000,000 | 21 | 0.283545841 | 4 | AMP | FALSE | 4.4122082 | 4 | AMP |
| C67 | 19 | 52,000,001 | 59,000,000 | 7 | 0.127081235 | 3 | GAIN | FALSE | 2.9945245 | 3 | GAIN |
| C67 | 20 | 1,000,001 | 15,000,000 | 14 | 0.140007783 | 3 | GAIN | FALSE | 3.1059103 | 3 | GAIN |
| C67 | 20 | 30,000,001 | 42,000,000 | 12 | 0.113710338 | 3 | GAIN | FALSE | 2.8803551 | 3 | GAIN |
| C67 | 20 | 15,000,001 | 25,000,000 | 10 | -0.031690239 | 1 | HETD | TRUE | 1.3146749 | 1 | HETD |
| C67 | 20 | 42,000,001 | 61,000,000 | 19 | 0.32915953 | 5 | HLAMP | FALSE | 4.8552856 | 5 | HLAMP |
| C67 | 21 | 16,000,001 | 48,000,000 | 32 | -0.025519833 | 1 | HETD | TRUE | 1.4249913 | 1 | HETD |
| C67 | 22 | 38,000,001 | 50,000,000 | 12 | 0.127149042 | 3 | GAIN | FALSE | 2.9951062 | 3 | GAIN |
| C67 | X | 4,000,001 | 151,000,000 | 147 | 0.040878764 | 3 | GAIN | TRUE | 2.642426 | 3 | GAIN |
| C68 | 1 | 1,000,001 | 93,000,000 | 92 | -0.103145482 | 1 | HETD | FALSE | 1.0182675 | 1 | HETD |
| C68 | 1 | 167,000,001 | 203,000,000 | 36 | -0.115903234 | 1 | HETD | FALSE | 0.9265471 | 1 | HETD |
| C68 | 1 | 115,000,001 | 167,000,000 | 52 | 0.627031332 | 5 | HLAMP | FALSE | 7.8820962 | 8 | HLAMP |
| C68 | 1 | 203,000,001 | 230,000,000 | 27 | 0.533045521 | 5 | HLAMP | FALSE | 6.7921419 | 7 | HLAMP |
| C68 | 2 | 1,000,001 | 20,000,000 | 19 | -0.123105168 | 1 | HETD | FALSE | 0.8751267 | 1 | HETD |
| C68 | 3 | 1,000,001 | 73,000,000 | 72 | -0.117706603 | 1 | HETD | FALSE | 0.9136473 | 1 | HETD |
| C68 | 3 | 182,000,001 | 197,000,000 | 15 | -0.126916768 | 1 | HETD | FALSE | 0.8480164 | 1 | HETD |
| C68 | 4 | 62,000,001 | 69,000,000 | 7 | -0.110177178 | 1 | HETD | FALSE | 0.9676136 | 1 | HETD |
| C68 | 4 | 104,000,001 | 191,000,000 | 87 | -0.093088455 | 1 | HETD | FALSE | 1.0911452 | 1 | HETD |
| C68 | 4 | 69,000,001 | 74,000,000 | 5 | 0.320126816 | 5 | HLAMP | FALSE | 4.5704523 | 5 | HLAMP |
| C68 | 5 | 164,000,001 | 180,000,000 | 16 | 0.127783828 | 3 | GAIN | FALSE | 2.8267374 | 3 | GAIN |
| C68 | 5 | 54,000,001 | 87,000,000 | 33 | -0.127387087 | 1 | HETD | FALSE | 0.8446761 | 1 | HETD |
| C68 | 6 | 28,000,001 | 34,000,000 | 6 | -4.432503062 | 4 | AMP | FALSE | 0.015625 | 4 | AMP |
| C68 | 6 | 82,000,001 | 124,000,000 | 42 | -0.104679067 | 1 | HETD | FALSE | 1.007199 | 1 | HETD |
| C68 | 6 | 142,000,001 | 170,000,000 | 28 | -0.08957405 | 1 | HETD | FALSE | 1.1167321 | 1 | HETD |
| C68 | 12 | 1,000,001 | 86,000,000 | 85 | 0.073920809 | 3 | GAIN | TRUE | 3.0649743 | 3 | GAIN |
| C68 | 12 | 86,000,001 | 132,000,000 | 46 | -0.104705035 | 1 | HETD | FALSE | 1.0070117 | 1 | HETD |
| C68 | 14 | 61,000,001 | 95,000,000 | 34 | -0.077608337 | 1 | HETD | FALSE | 1.2043185 | 1 | HETD |
| C68 | 15 | 25,000,001 | 102,000,000 | 77 | -0.102158679 | 1 | HETD | FALSE | 1.0253959 | 1 | HETD |
| C68 | 19 | 1,000,001 | 59,000,000 | 58 | 0.07123451 | 3 | GAIN | TRUE | 3.0033529 | 3 | GAIN |
| C68 | 22 | 22,000,001 | 50,000,000 | 28 | -0.10767843 | 1 | HETD | FALSE | 0.9855855 | 1 | HETD |
| C69 | 6 | 28,000,001 | 34,000,000 | 6 | -5.377850628 | 1 | HETD | FALSE | 0.015625 | 1 | HETD |
| C70 | 1 | 1,000,001 | 17,000,000 | 16 | 0.144257963 | 4 | AMP | FALSE | 4.0437902 | 4 | AMP |
| C70 | 1 | 33,000,001 | 92,000,000 | 59 | 0.149230932 | 4 | AMP | FALSE | 4.0944026 | 4 | AMP |
| C70 | 1 | 151,000,001 | 248,000,000 | 97 | 0.043937277 | 3 | GAIN | FALSE | 3.0591652 | 3 | GAIN |
| C70 | 1 | 18,000,001 | 33,000,000 | 15 | -0.19733327 | 1 | HETD | FALSE | 0.9535036 | 1 | HETD |
| C70 | 1 | 92,000,001 | 151,000,000 | 59 | 0.260379044 | 5 | HLAMP | FALSE | 5.2723574 | 5 | HLAMP |
| C70 | 2 | 1,000,001 | 149,000,000 | 148 | 0.032971352 | 3 | GAIN | FALSE | 2.9556298 | 3 | GAIN |
| C70 | 3 | 160,000,001 | 197,000,000 | 37 | 0.051688878 | 3 | GAIN | FALSE | 3.1328286 | 3 | GAIN |
| C70 | 4 | 2,000,001 | 191,000,000 | 189 | 0.010091652 | 3 | GAIN | FALSE | 2.7421272 | 3 | GAIN |
| C70 | 6 | 34,000,001 | 39,000,000 | 5 | 0.037163824 | 3 | GAIN | FALSE | 2.9951203 | 3 | GAIN |
| C70 | 6 | 5,000,001 | 28,000,000 | 23 | -0.202055211 | 1 | HETD | FALSE | 0.9157052 | 1 | HETD |
| C70 | 6 | 28,000,001 | 34,000,000 | 6 | -4.14336245 | 1 | HETD | TRUE | 0.015625 | 1 | HETD |
| C70 | 7 | 1,000,001 | 159,000,000 | 158 | 0.051312335 | 3 | GAIN | FALSE | 3.1292411 | 3 | GAIN |
| C70 | 8 | 1,000,001 | 145,000,000 | 144 | 0.040471924 | 3 | GAIN | FALSE | 3.0263618 | 3 | GAIN |
| C70 | 10 | 1,000,001 | 135,000,000 | 134 | 0.038264601 | 3 | GAIN | FALSE | 3.005508 | 3 | GAIN |
| C70 | 11 | 2,000,001 | 134,000,000 | 132 | 0.045962821 | 3 | GAIN | FALSE | 3.0783757 | 3 | GAIN |
| C70 | 12 | 1,000,001 | 132,000,000 | 131 | 0.048913133 | 3 | GAIN | FALSE | 3.1064052 | 3 | GAIN |
| C70 | 13 | 20,000,001 | 114,000,000 | 94 | 0.045139042 | 3 | GAIN | FALSE | 3.0705596 | 3 | GAIN |
| C70 | 14 | 21,000,001 | 106,000,000 | 85 | 0.02157839 | 3 | GAIN | FALSE | 2.8488928 | 3 | GAIN |
| C70 | 16 | 1,000,001 | 90,000,000 | 89 | 0.038880631 | 3 | GAIN | FALSE | 3.0113248 | 3 | GAIN |
| C70 | 17 | 26,000,001 | 81,000,000 | 55 | 0.043892554 | 3 | GAIN | FALSE | 3.0587413 | 3 | GAIN |
| C70 | 17 | 1,000,001 | 18,000,000 | 17 | -0.189384021 | 1 | HETD | FALSE | 1.0174163 | 1 | HETD |
| C70 | 18 | 1,000,001 | 22,000,000 | 21 | 0.025423172 | 3 | GAIN | FALSE | 2.8848191 | 3 | GAIN |
| C70 | 18 | 22,000,001 | 78,000,000 | 56 | -0.213436632 | 1 | HETD | FALSE | 0.8251056 | 1 | HETD |
| C70 | 19 | 1,000,001 | 59,000,000 | 58 | 0.083295441 | 4 | AMP | FALSE | 3.4373163 | 4 | AMP |
| C70 | 20 | 24,000,001 | 52,000,000 | 28 | 0.005698645 | 3 | GAIN | FALSE | 2.7015198 | 3 | GAIN |
| C70 | 20 | 1,000,001 | 24,000,000 | 23 | -0.21739106 | 1 | HETD | FALSE | 0.7937941 | 1 | HETD |
| C70 | 21 | 16,000,001 | 48,000,000 | 32 | 0.037342718 | 3 | GAIN | FALSE | 2.996808 | 3 | GAIN |
| C70 | 22 | 22,000,001 | 50,000,000 | 28 | 0.008204607 | 3 | GAIN | FALSE | 2.7246688 | 3 | GAIN |
| C70 | X | 4,000,001 | 151,000,000 | 147 | -0.006378155 | 3 | GAIN | FALSE | 1.2952606 | 3 | GAIN |
| C71 | 17 | 27,000,001 | 81,000,000 | 54 | 0.043410694 | 3 | GAIN | FALSE | 2.9635313 | 3 | GAIN |
| C71 | 19 | 1,000,001 | 59,000,000 | 58 | 0.053502346 | 3 | GAIN | FALSE | 3.1889764 | 3 | GAIN |
| C72 | 1 | 1,000,001 | 248,000,000 | 247 | 0.045567523 | 3 | GAIN | FALSE | 3.0932102 | 3 | GAIN |
| C72 | 2 | 1,000,001 | 243,000,000 | 242 | 0.028266724 | 3 | GAIN | FALSE | 2.7339299 | 3 | GAIN |
| C72 | 3 | 94,000,001 | 197,000,000 | 103 | 0.095341944 | 4 | AMP | FALSE | 4.1512164 | 4 | AMP |
| C72 | 6 | 1,000,001 | 28,000,000 | 27 | -0.040080954 | 1 | HETD | TRUE | 0.8613549 | 1 | HETD |
| C72 | 6 | 28,000,001 | 34,000,000 | 6 | -4.004035619 | 1 | HETD | FALSE | 0.015625 | 1 | HETD |
| C72 | 6 | 34,000,001 | 170,000,000 | 136 | -0.032319254 | 1 | HETD | TRUE | 1.132244 | 1 | HETD |
| C72 | 7 | 39,000,001 | 78,000,000 | 39 | 0.035610042 | 3 | GAIN | FALSE | 2.8859001 | 3 | GAIN |
| C72 | 7 | 120,000,001 | 159,000,000 | 39 | 0.026114223 | 3 | GAIN | FALSE | 2.6895302 | 3 | GAIN |
| C72 | 8 | 1,000,001 | 58,000,000 | 57 | 0.036875285 | 3 | GAIN | FALSE | 2.9121626 | 3 | GAIN |
| C72 | 10 | 1,000,001 | 135,000,000 | 134 | -0.022351674 | 1 | HETD | TRUE | 1.4822643 | 1 | HETD |
| C72 | 13 | 20,000,001 | 114,000,000 | 94 | -0.046777944 | 1 | HETD | TRUE | 0.6287933 | 1 | HETD |
| C72 | 14 | 21,000,001 | 106,000,000 | 85 | -0.031857592 | 1 | HETD | TRUE | 1.1484023 | 1 | HETD |
| C72 | 18 | 1,000,001 | 23,000,000 | 22 | 0.090901329 | 4 | AMP | FALSE | 4.0553366 | 4 | AMP |
| C72 | 18 | 23,000,001 | 78,000,000 | 55 | -0.230144484 | 1 | HETD | FALSE | 0.015625 | 1 | HETD |
| C72 | 19 | 1,000,001 | 59,000,000 | 58 | 0.036604059 | 3 | GAIN | FALSE | 2.9065309 | 3 | GAIN |
| C72 | 21 | 16,000,001 | 48,000,000 | 32 | -0.047998464 | 1 | HETD | TRUE | 0.5865253 | 1 | HETD |
| C72 | X | 4,000,001 | 151,000,000 | 147 | -0.161457773 | 1 | HETD | FALSE | 0.015625 | 1 | HETD |
| C74 | 6 | 28,000,001 | 34,000,000 | 6 | -6.100744717 | 1 | HETD | TRUE | 0.015625 | 1 | HETD |
| C74 | 19 | 1,000,001 | 59,000,000 | 58 | 0.049107896 | 3 | GAIN | FALSE | 2.9056932 | 3 | GAIN |
| C75 | 1 | 1,000,001 | 17,000,000 | 16 | 0.130367695 | 3 | GAIN | FALSE | 2.805829 | 3 | GAIN |
| C75 | 1 | 31,000,001 | 47,000,000 | 16 | 0.140861858 | 3 | GAIN | FALSE | 2.8858916 | 3 | GAIN |
| C75 | 1 | 18,000,001 | 29,000,000 | 11 | -0.137288303 | 1 | HETD | FALSE | 0.9488175 | 1 | HETD |
| C75 | 1 | 47,000,001 | 68,000,000 | 21 | -0.113704567 | 1 | HETD | FALSE | 1.0989573 | 1 | HETD |
| C75 | 4 | 174,000,001 | 191,000,000 | 17 | -0.159514463 | 1 | HETD | FALSE | 0.8095493 | 1 | HETD |
| C75 | 5 | 1,000,001 | 46,000,000 | 45 | 0.14780555 | 3 | GAIN | FALSE | 2.9391878 | 3 | GAIN |
| C75 | 5 | 50,000,001 | 152,000,000 | 102 | -0.136558299 | 1 | HETD | FALSE | 0.9534282 | 1 | HETD |
| C75 | 6 | 1,000,001 | 49,000,000 | 48 | -0.029732502 | 1 | HETD | TRUE | 1.07347 | 1 | HETD |
| C75 | 7 | 1,000,001 | 159,000,000 | 158 | -0.032775146 | 1 | HETD | TRUE | 1.0181118 | 1 | HETD |
| C75 | 8 | 1,000,001 | 25,000,000 | 24 | -0.107705841 | 1 | HETD | FALSE | 1.1375398 | 1 | HETD |
| C75 | 8 | 25,000,001 | 129,000,000 | 104 | -0.039808284 | 1 | HETD | TRUE | 0.8905961 | 1 | HETD |
| C75 | 8 | 129,000,001 | 145,000,000 | 16 | -0.134457735 | 1 | HETD | FALSE | 0.9667083 | 1 | HETD |
| C75 | 9 | 4,000,001 | 38,000,000 | 34 | -0.145591013 | 1 | HETD | FALSE | 0.8965419 | 1 | HETD |
| C75 | 10 | 1,000,001 | 37,000,000 | 36 | -0.118517398 | 1 | HETD | FALSE | 1.068118 | 1 | HETD |
| C75 | 13 | 20,000,001 | 114,000,000 | 94 | 0.14440656 | 3 | GAIN | FALSE | 2.9130668 | 3 | GAIN |
| C75 | 14 | 21,000,001 | 106,000,000 | 85 | -0.133062649 | 1 | HETD | FALSE | 0.9755389 | 1 | HETD |
| C75 | 17 | 1,000,001 | 18,000,000 | 17 | -0.140763755 | 1 | HETD | FALSE | 0.9268987 | 1 | HETD |
| C75 | 18 | 1,000,001 | 24,000,000 | 23 | -0.011176558 | 1 | HETD | TRUE | 1.4136176 | 1 | HETD |
| C75 | 18 | 24,000,001 | 78,000,000 | 54 | -0.153367139 | 1 | HETD | FALSE | 0.8478538 | 1 | HETD |
| C75 | 19 | 1,000,001 | 20,000,000 | 19 | -0.073127326 | 1 | HETD | TRUE | 0.2948757 | 1 | HETD |
| C75 | X | 4,000,001 | 72,000,000 | 68 | -0.015076163 | 1 | HETD | TRUE | 0.6708853 | 1 | HETD |
| C76 | 6 | 28,000,001 | 34,000,000 | 6 | -4.511577177 | 1 | HETD | TRUE | 0.015625 | 1 | HETD |
| C76 | 19 | 1,000,001 | 59,000,000 | 58 | 0.034337585 | 3 | GAIN | FALSE | 2.9825909 | 3 | GAIN |
| C77 | 1 | 204,000,001 | 248,000,000 | 44 | 0.575289075 | 4 | AMP | FALSE | 4.5831728 | 4 | AMP |
| C77 | 1 | 1,000,001 | 12,000,000 | 11 | 0.282238759 | 3 | GAIN | FALSE | 3.2518515 | 3 | GAIN |
| C77 | 1 | 12,000,001 | 24,000,000 | 12 | 0.069220508 | 3 | GAIN | TRUE | 2.647453 | 3 | GAIN |
| C77 | 1 | 32,000,001 | 67,000,000 | 35 | 0.149496388 | 3 | GAIN | TRUE | 3.0763907 | 3 | GAIN |
| C77 | 1 | 67,000,001 | 90,000,000 | 23 | 0.203701326 | 3 | GAIN | FALSE | 2.9386707 | 3 | GAIN |
| C77 | 1 | 150,000,001 | 204,000,000 | 54 | 0.127917998 | 3 | GAIN | TRUE | 2.9587356 | 3 | GAIN |
| C77 | 1 | 24,000,001 | 32,000,000 | 8 | -0.409376259 | 1 | HETD | TRUE | 0.5310723 | 1 | HETD |
| C77 | 1 | 90,000,001 | 120,000,000 | 30 | -0.407174707 | 1 | HETD | FALSE | 1.0063006 | 1 | HETD |
| C77 | 2 | 1,000,001 | 40,000,000 | 39 | 0.1687203 | 3 | GAIN | TRUE | 3.1827008 | 3 | GAIN |
| C77 | 2 | 44,000,001 | 63,000,000 | 19 | 0.21641147 | 3 | GAIN | FALSE | 2.9882053 | 3 | GAIN |
| C77 | 2 | 163,000,001 | 233,000,000 | 70 | 0.207055592 | 3 | GAIN | FALSE | 2.9517008 | 3 | GAIN |
| C77 | 2 | 40,000,001 | 44,000,000 | 4 | -0.390616896 | 1 | HETD | FALSE | 1.0486112 | 1 | HETD |
| C77 | 2 | 99,000,001 | 163,000,000 | 64 | -0.426486623 | 1 | HETD | FALSE | 0.9575619 | 1 | HETD |
| C77 | 2 | 233,000,001 | 243,000,000 | 10 | -0.550489147 | 1 | HETD | FALSE | 0.6596841 | 1 | HETD |
| C77 | 3 | 1,000,001 | 44,000,000 | 43 | 0.186067265 | 3 | GAIN | FALSE | 2.8706652 | 3 | GAIN |
| C77 | 3 | 88,000,001 | 197,000,000 | 109 | 0.248130563 | 3 | GAIN | FALSE | 3.1137429 | 3 | GAIN |
| C77 | 3 | 44,000,001 | 88,000,000 | 44 | -0.387526643 | 1 | HETD | FALSE | 1.0565617 | 1 | HETD |
| C77 | 4 | 2,000,001 | 59,000,000 | 57 | 0.15729704 | 3 | GAIN | TRUE | 3.1193584 | 3 | GAIN |
| C77 | 4 | 126,000,001 | 147,000,000 | 21 | 0.15997324 | 3 | GAIN | TRUE | 3.134153 | 3 | GAIN |
| C77 | 4 | 154,000,001 | 191,000,000 | 37 | 0.22016969 | 3 | GAIN | FALSE | 3.0029358 | 3 | GAIN |
| C77 | 4 | 60,000,001 | 126,000,000 | 66 | -0.383482769 | 1 | HETD | FALSE | 1.0669914 | 1 | HETD |
| C77 | 5 | 1,000,001 | 46,000,000 | 45 | 0.230349346 | 3 | GAIN | FALSE | 3.0430287 | 3 | GAIN |
| C77 | 5 | 50,000,001 | 180,000,000 | 130 | 0.10614196 | 3 | GAIN | TRUE | 2.8417734 | 3 | GAIN |
| C77 | 6 | 34,000,001 | 46,000,000 | 12 | 0.42811254 | 4 | AMP | FALSE | 3.8807843 | 4 | AMP |
| C77 | 6 | 46,000,001 | 57,000,000 | 11 | 0.123516336 | 3 | GAIN | TRUE | 2.934951 | 3 | GAIN |
| C77 | 6 | 1,000,001 | 28,000,000 | 27 | -0.415903012 | 1 | HETD | FALSE | 0.9841916 | 1 | HETD |
| C77 | 6 | 63,000,001 | 170,000,000 | 107 | -0.388331667 | 1 | HETD | FALSE | 1.0544889 | 1 | HETD |
| C77 | 6 | 28,000,001 | 34,000,000 | 6 | -5.294526333 | 5 | HLAMP | FALSE | 0.015625 | 0 | HOMD |
| C77 | 8 | 31,000,001 | 52,000,000 | 21 | 0.535763558 | 4 | AMP | FALSE | 4.3874485 | 4 | AMP |
| C77 | 8 | 69,000,001 | 103,000,000 | 34 | 0.690081434 | 4 | AMP | FALSE | 5.1829753 | 4 | AMP |
| C77 | 8 | 1,000,001 | 31,000,000 | 30 | 0.15594235 | 3 | GAIN | TRUE | 3.1118798 | 3 | GAIN |
| C77 | 8 | 52,000,001 | 69,000,000 | 17 | 0.253425254 | 3 | GAIN | FALSE | 3.1349683 | 3 | GAIN |
| C77 | 8 | 103,000,001 | 145,000,000 | 42 | 0.251098186 | 3 | GAIN | FALSE | 3.1256299 | 3 | GAIN |
| C77 | 9 | 33,000,001 | 36,000,000 | 3 | 0.220540463 | 3 | GAIN | TRUE | 3.4764283 | 3 | GAIN |
| C77 | 9 | 131,000,001 | 141,000,000 | 10 | 0.075584236 | 3 | GAIN | TRUE | 2.6805921 | 3 | GAIN |
| C77 | 9 | 1,000,001 | 33,000,000 | 32 | -0.370146496 | 1 | HETD | FALSE | 1.1015955 | 1 | HETD |
| C77 | 10 | 1,000,001 | 16,000,000 | 15 | -0.457527649 | 1 | HETD | FALSE | 0.8805761 | 1 | HETD |
| C77 | 11 | 91,000,001 | 120,000,000 | 29 | -0.815255426 | 1 | HETD | TRUE | 0.015625 | 1 | HETD |
| C77 | 12 | 1,000,001 | 39,000,000 | 38 | 0.15233994 | 3 | GAIN | TRUE | 3.0920267 | 3 | GAIN |
| C77 | 12 | 74,000,001 | 83,000,000 | 9 | 0.167524305 | 3 | GAIN | TRUE | 3.1760454 | 3 | GAIN |
| C77 | 12 | 39,000,001 | 53,000,000 | 14 | -0.367503479 | 1 | HETD | FALSE | 1.1084915 | 1 | HETD |
| C77 | 12 | 119,000,001 | 132,000,000 | 13 | -0.223695637 | 1 | HETD | TRUE | 1.2700179 | 1 | HETD |
| C77 | 12 | 53,000,001 | 74,000,000 | 21 | 1.786418577 | 5 | HLAMP | FALSE | 14.108178 | 14 | HLAMP |
| C77 | 12 | 93,000,001 | 119,000,000 | 26 | 1.015249026 | 5 | HLAMP | FALSE | 7.1655403 | 7 | HLAMP |
| C77 | 16 | 1,000,001 | 90,000,000 | 89 | 0.161371211 | 3 | GAIN | TRUE | 3.1418923 | 3 | GAIN |
| C77 | 17 | 26,000,001 | 81,000,000 | 55 | 0.219386307 | 3 | GAIN | FALSE | 2.9998622 | 3 | GAIN |
| C77 | 18 | 23,000,001 | 35,000,000 | 12 | 0.584072202 | 4 | AMP | FALSE | 4.6273985 | 4 | AMP |
| C77 | 18 | 1,000,001 | 23,000,000 | 22 | 0.148724546 | 3 | GAIN | TRUE | 3.0721519 | 3 | GAIN |
| C77 | 18 | 35,000,001 | 78,000,000 | 43 | -0.46310078 | 1 | HETD | FALSE | 0.8669286 | 1 | HETD |
| C77 | 20 | 30,000,001 | 61,000,000 | 31 | 0.119005029 | 3 | GAIN | TRUE | 2.9106492 | 3 | GAIN |
| C77 | 20 | 1,000,001 | 25,000,000 | 24 | -0.432429083 | 1 | HETD | FALSE | 0.9426954 | 1 | HETD |
| C77 | 21 | 16,000,001 | 48,000,000 | 32 | -0.368796133 | 1 | HETD | FALSE | 1.1051172 | 1 | HETD |
| C77 | 22 | 38,000,001 | 50,000,000 | 12 | 0.084162501 | 3 | GAIN | TRUE | 2.7254953 | 3 | GAIN |
| C77 | X | 4,000,001 | 58,000,000 | 54 | 0.589242821 | 4 | AMP | FALSE | 2.3267801 | 4 | AMP |
| C78 | 1 | 11,000,001 | 36,000,000 | 25 | -0.075246645 | 1 | HETD | FALSE | 1.0498054 | 1 | HETD |
| C78 | 3 | 68,000,001 | 81,000,000 | 13 | 0.130553802 | 4 | AMP | FALSE | 3.6049777 | 4 | AMP |
| C78 | 3 | 159,000,001 | 197,000,000 | 38 | 0.057521694 | 3 | GAIN | FALSE | 2.6562326 | 3 | GAIN |
| C78 | 3 | 1,000,001 | 68,000,000 | 67 | -0.085913932 | 1 | HETD | FALSE | 0.9270391 | 1 | HETD |
| C78 | 5 | 95,000,001 | 108,000,000 | 13 | -0.087875998 | 1 | HETD | FALSE | 0.904557 | 1 | HETD |
| C78 | 6 | 132,000,001 | 137,000,000 | 5 | 0.137712455 | 4 | AMP | FALSE | 3.700585 | 4 | AMP |
| C78 | 6 | 23,000,001 | 34,000,000 | 11 | -0.829962259 | 3 | GAIN | TRUE | 0.015625 | 3 | GAIN |
| C78 | 6 | 1,000,001 | 23,000,000 | 22 | -0.070267905 | 1 | HETD | FALSE | 1.1074155 | 1 | HETD |
| C78 | 6 | 69,000,001 | 132,000,000 | 63 | -0.096089261 | 1 | HETD | FALSE | 0.8107775 | 1 | HETD |
| C78 | 6 | 158,000,001 | 170,000,000 | 12 | -0.093065372 | 1 | HETD | FALSE | 0.8452424 | 1 | HETD |
| C78 | 7 | 88,000,001 | 148,000,000 | 60 | 0.152664547 | 4 | AMP | FALSE | 3.9018143 | 4 | AMP |
| C78 | 7 | 1,000,001 | 81,000,000 | 80 | 0.059149054 | 3 | GAIN | FALSE | 2.6768543 | 3 | GAIN |
| C78 | 7 | 148,000,001 | 159,000,000 | 11 | -0.059434708 | 1 | HETD | FALSE | 1.2334579 | 1 | HETD |
| C78 | 8 | 87,000,001 | 145,000,000 | 58 | 0.094604727 | 3 | GAIN | FALSE | 3.1319682 | 3 | GAIN |
| C78 | 9 | 1,000,001 | 141,000,000 | 140 | -0.090299725 | 1 | HETD | FALSE | 0.8768272 | 1 | HETD |
| C78 | 14 | 75,000,001 | 106,000,000 | 31 | -0.049345941 | 1 | HETD | FALSE | 1.3516931 | 1 | HETD |
| C78 | 16 | 1,000,001 | 14,000,000 | 13 | 0.109940576 | 3 | GAIN | TRUE | 4.2216685 | 3 | GAIN |
| C78 | 17 | 26,000,001 | 44,000,000 | 18 | 0.100733296 | 3 | GAIN | FALSE | 3.2117754 | 3 | GAIN |
| C78 | 17 | 1,000,001 | 18,000,000 | 17 | -0.114860166 | 1 | HETD | FALSE | 0.5984441 | 1 | HETD |
| C78 | 18 | 35,000,001 | 78,000,000 | 43 | -0.097720949 | 1 | HETD | FALSE | 0.7922103 | 1 | HETD |
| C78 | 20 | 30,000,001 | 61,000,000 | 31 | 0.09786089 | 3 | GAIN | FALSE | 3.1743283 | 3 | GAIN |
| C78 | 20 | 1,000,001 | 25,000,000 | 24 | -0.067591705 | 1 | HETD | FALSE | 1.1384646 | 1 | HETD |
| C79 | 19 | 1,000,001 | 59,000,000 | 58 | 0.032583267 | 3 | GAIN | FALSE | 2.8153299 | 3 | GAIN |
| C80 | 3 | 94,000,001 | 197,000,000 | 103 | 0.047320397 | 3 | GAIN | FALSE | 2.8582622 | 3 | GAIN |
| C80 | 3 | 1,000,001 | 90,000,000 | 89 | -0.052147655 | 1 | HETD | FALSE | 0.8646366 | 1 | HETD |
| C80 | 4 | 2,000,001 | 127,000,000 | 125 | -0.025964183 | 1 | HETD | FALSE | 1.3761732 | 1 | HETD |
| C80 | 5 | 1,000,001 | 46,000,000 | 45 | 0.109800634 | 4 | AMP | FALSE | 4.1826837 | 4 | AMP |
| C80 | 5 | 50,000,001 | 180,000,000 | 130 | -0.046513931 | 1 | HETD | FALSE | 0.973918 | 1 | HETD |
| C80 | 6 | 28,000,001 | 34,000,000 | 6 | -4.512047495 | 1 | HETD | TRUE | 0.015625 | 1 | HETD |
| C80 | 6 | 34,000,001 | 170,000,000 | 136 | -0.047838325 | 1 | HETD | FALSE | 0.9481894 | 1 | HETD |
| C80 | 7 | 1,000,001 | 159,000,000 | 158 | 0.059469332 | 3 | GAIN | FALSE | 3.1113163 | 3 | GAIN |
| C80 | 8 | 47,000,001 | 110,000,000 | 63 | 0.092082896 | 4 | AMP | FALSE | 3.8012684 | 4 | AMP |
| C80 | 8 | 110,000,001 | 145,000,000 | 35 | 0.058755198 | 3 | GAIN | FALSE | 3.0963824 | 3 | GAIN |
| C80 | 8 | 1,000,001 | 43,000,000 | 42 | -0.056226154 | 1 | HETD | FALSE | 0.7857889 | 1 | HETD |
| C80 | 9 | 1,000,001 | 29,000,000 | 28 | -0.03141757 | 1 | HETD | FALSE | 1.2688657 | 1 | HETD |
| C80 | 10 | 1,000,001 | 135,000,000 | 134 | -0.052346747 | 1 | HETD | FALSE | 0.8607825 | 1 | HETD |
| C80 | 14 | 21,000,001 | 106,000,000 | 85 | -0.023155255 | 1 | HETD | FALSE | 1.4316036 | 1 | HETD |
| C80 | 15 | 25,000,001 | 102,000,000 | 77 | -0.029435307 | 1 | HETD | FALSE | 1.3078242 | 1 | HETD |
| C80 | 19 | 21,000,001 | 59,000,000 | 38 | 0.142914813 | 4 | AMP | FALSE | 4.908224 | 4 | AMP |
| C80 | 20 | 1,000,001 | 61,000,000 | 60 | 0.066353873 | 3 | GAIN | FALSE | 3.2556655 | 3 | GAIN |
| C80 | 21 | 16,000,001 | 48,000,000 | 32 | -0.065641182 | 1 | HETD | FALSE | 0.6046215 | 1 | HETD |
| C80 | 22 | 33,000,001 | 50,000,000 | 17 | -0.051704748 | 1 | HETD | FALSE | 0.8732125 | 1 | HETD |
| C81 | 1 | 225,000,001 | 243,000,000 | 18 | 0.237901855 | 3 | GAIN | FALSE | 3.0130029 | 3 | GAIN |
| C81 | 1 | 15,000,001 | 33,000,000 | 18 | -0.162529695 | 1 | HETD | FALSE | 1.0449579 | 1 | HETD |
| C81 | 1 | 50,000,001 | 70,000,000 | 20 | -0.196290202 | 1 | HETD | FALSE | 0.902666 | 1 | HETD |
| C81 | 1 | 192,000,001 | 211,000,000 | 19 | -0.174189557 | 1 | HETD | FALSE | 0.9954377 | 1 | HETD |
| C81 | 2 | 1,000,001 | 3,000,000 | 2 | 0.200758228 | 3 | GAIN | TRUE | 3.4893231 | 3 | GAIN |
| C81 | 2 | 58,000,001 | 66,000,000 | 8 | 0.319868574 | 3 | GAIN | FALSE | 3.4877012 | 3 | GAIN |
| C81 | 2 | 4,000,001 | 58,000,000 | 54 | -0.087340758 | 1 | HETD | TRUE | 0.8443137 | 1 | HETD |
| C81 | 2 | 195,000,001 | 213,000,000 | 18 | -0.066449091 | 1 | HETD | TRUE | 1.0188647 | 1 | HETD |
| C81 | 2 | 233,000,001 | 243,000,000 | 10 | -0.182421574 | 1 | HETD | FALSE | 0.9607161 | 1 | HETD |
| C81 | 3 | 47,000,001 | 74,000,000 | 27 | -0.185768627 | 1 | HETD | FALSE | 0.9466551 | 1 | HETD |
| C81 | 4 | 2,000,001 | 19,000,000 | 17 | 0.366597466 | 4 | AMP | FALSE | 3.7706428 | 4 | AMP |
| C81 | 4 | 19,000,001 | 44,000,000 | 25 | -0.173635562 | 1 | HETD | FALSE | 0.9977815 | 1 | HETD |
| C81 | 4 | 81,000,001 | 191,000,000 | 110 | -0.064299475 | 1 | HETD | TRUE | 1.0369686 | 1 | HETD |
| C81 | 5 | 151,000,001 | 168,000,000 | 17 | 0.120636717 | 3 | GAIN | TRUE | 2.6999673 | 3 | GAIN |
| C81 | 5 | 64,000,001 | 91,000,000 | 27 | -0.209140287 | 1 | HETD | FALSE | 0.8493752 | 1 | HETD |
| C81 | 6 | 1,000,001 | 34,000,000 | 33 | -0.138345798 | 1 | HETD | TRUE | 0.4286332 | 1 | HETD |
| C81 | 6 | 145,000,001 | 170,000,000 | 25 | -0.149985462 | 1 | HETD | FALSE | 1.0986829 | 1 | HETD |
| C81 | 7 | 4,000,001 | 49,000,000 | 45 | 0.245887172 | 3 | GAIN | FALSE | 3.058072 | 3 | GAIN |
| C81 | 8 | 65,000,001 | 145,000,000 | 80 | 0.157331665 | 3 | GAIN | TRUE | 3.0560489 | 3 | GAIN |
| C81 | 8 | 1,000,001 | 41,000,000 | 40 | -0.171432566 | 1 | HETD | FALSE | 1.0071107 | 1 | HETD |
| C81 | 9 | 1,000,001 | 38,000,000 | 37 | -0.183512252 | 1 | HETD | FALSE | 0.9561306 | 1 | HETD |
| C81 | 9 | 115,000,001 | 119,000,000 | 4 | -0.163849761 | 1 | HETD | FALSE | 1.0393314 | 1 | HETD |
| C81 | 10 | 1,000,001 | 20,000,000 | 19 | 0.142151628 | 3 | GAIN | TRUE | 2.9076452 | 3 | GAIN |
| C81 | 10 | 20,000,001 | 37,000,000 | 17 | -0.025189455 | 1 | HETD | TRUE | 1.371106 | 1 | HETD |
| C81 | 10 | 78,000,001 | 116,000,000 | 38 | -0.180896646 | 1 | HETD | FALSE | 0.9671331 | 1 | HETD |
| C81 | 11 | 2,000,001 | 15,000,000 | 13 | -0.204113937 | 1 | HETD | FALSE | 0.8701635 | 1 | HETD |
| C81 | 12 | 1,000,001 | 24,000,000 | 23 | 0.117768639 | 3 | GAIN | TRUE | 2.6725157 | 3 | GAIN |
| C81 | 12 | 74,000,001 | 96,000,000 | 22 | -0.168138894 | 1 | HETD | FALSE | 1.0210853 | 1 | HETD |
| C81 | 12 | 104,000,001 | 132,000,000 | 28 | -0.162350801 | 1 | HETD | FALSE | 1.0457208 | 1 | HETD |
| C81 | 13 | 20,000,001 | 26,000,000 | 6 | 0.16945896 | 3 | GAIN | TRUE | 3.1757354 | 3 | GAIN |
| C81 | 13 | 32,000,001 | 108,000,000 | 76 | -0.160828758 | 1 | HETD | FALSE | 1.0522154 | 1 | HETD |
| C81 | 14 | 36,000,001 | 106,000,000 | 70 | -0.167090054 | 1 | HETD | FALSE | 1.0255421 | 1 | HETD |
| C81 | 15 | 86,000,001 | 102,000,000 | 16 | 0.305520972 | 3 | GAIN | FALSE | 3.40265 | 3 | GAIN |
| C81 | 16 | 1,000,001 | 32,000,000 | 31 | 0.189997166 | 3 | GAIN | TRUE | 3.3807396 | 3 | GAIN |
| C81 | 16 | 47,000,001 | 67,000,000 | 20 | -0.029834933 | 1 | HETD | TRUE | 1.3309415 | 1 | HETD |
| C81 | 17 | 26,000,001 | 39,000,000 | 13 | 0.139964502 | 3 | GAIN | TRUE | 2.8863918 | 3 | GAIN |
| C81 | 17 | 65,000,001 | 71,000,000 | 6 | 0.161029292 | 3 | GAIN | TRUE | 3.0924349 | 3 | GAIN |
| C81 | 17 | 1,000,001 | 18,000,000 | 17 | -0.149920541 | 1 | HETD | FALSE | 1.0989621 | 1 | HETD |
| C81 | 17 | 71,000,001 | 81,000,000 | 10 | -0.081585847 | 1 | HETD | TRUE | 0.8921443 | 1 | HETD |
| C81 | 18 | 32,000,001 | 39,000,000 | 7 | 0.247624177 | 3 | GAIN | FALSE | 3.0679087 | 3 | GAIN |
| C81 | 18 | 1,000,001 | 3,000,000 | 2 | -0.208091447 | 1 | HETD | FALSE | 0.8537071 | 1 | HETD |
| C81 | 18 | 39,000,001 | 78,000,000 | 39 | -0.174991695 | 1 | HETD | FALSE | 0.9920457 | 1 | HETD |
| C81 | 21 | 25,000,001 | 35,000,000 | 10 | -0.14601228 | 1 | HETD | FALSE | 1.115797 | 1 | HETD |
| C81 | X | 4,000,001 | 49,000,000 | 45 | -0.1621041 | 1 | HETD | FALSE | 1.046773 | 1 | HETD |
| C81 | X | 69,000,001 | 151,000,000 | 82 | -0.079752182 | 1 | HETD | TRUE | 0.9074245 | 1 | HETD |
| C82 | 12 | 1,000,001 | 65,000,000 | 64 | 0.032248562 | 3 | GAIN | FALSE | 2.9346802 | 3 | GAIN |
| C82 | 17 | 1,000,001 | 81,000,000 | 80 | 0.027927691 | 3 | GAIN | FALSE | 2.8143742 | 3 | GAIN |
| C82 | 19 | 1,000,001 | 59,000,000 | 58 | 0.048851097 | 3 | GAIN | FALSE | 3.4003115 | 3 | GAIN |
| C82 | 21 | 16,000,001 | 48,000,000 | 32 | 0.021993886 | 3 | GAIN | FALSE | 2.6497453 | 3 | GAIN |
| C83 | 19 | 1,000,001 | 59,000,000 | 58 | 0.058172349 | 3 | GAIN | FALSE | 2.9863984 | 3 | GAIN |
| C84 | 1 | 192,000,001 | 221,000,000 | 29 | -0.090638759 | 1 | HETD | FALSE | 0.9621325 | 1 | HETD |
| C84 | 2 | 1,000,001 | 65,000,000 | 64 | 0.057928534 | 3 | GAIN | TRUE | 3.3198191 | 3 | GAIN |
| C84 | 2 | 65,000,001 | 243,000,000 | 178 | -0.022710905 | 1 | HETD | TRUE | 1.0262104 | 1 | HETD |
| C84 | 3 | 151,000,001 | 197,000,000 | 46 | 0.098866448 | 3 | GAIN | FALSE | 2.9363967 | 3 | GAIN |
| C84 | 3 | 1,000,001 | 85,000,000 | 84 | -0.078375851 | 1 | HETD | FALSE | 1.0821899 | 1 | HETD |
| C84 | 4 | 2,000,001 | 24,000,000 | 22 | -0.029635842 | 1 | HETD | TRUE | 0.8351583 | 1 | HETD |
| C84 | 6 | 28,000,001 | 45,000,000 | 17 | -0.094433047 | 4 | AMP | FALSE | 0.9251916 | 4 | AMP |
| C84 | 6 | 1,000,001 | 28,000,000 | 27 | -0.087728843 | 1 | HETD | FALSE | 0.9905291 | 1 | HETD |
| C84 | 7 | 1,000,001 | 33,000,000 | 32 | 0.063523305 | 3 | GAIN | TRUE | 3.4837563 | 3 | GAIN |
| C84 | 7 | 33,000,001 | 95,000,000 | 62 | 0.083614276 | 3 | GAIN | FALSE | 2.7677284 | 3 | GAIN |
| C84 | 8 | 40,000,001 | 103,000,000 | 63 | 0.096092146 | 3 | GAIN | FALSE | 2.9055838 | 3 | GAIN |
| C84 | 8 | 1,000,001 | 40,000,000 | 39 | -0.114988566 | 1 | HETD | FALSE | 0.7267445 | 1 | HETD |
| C84 | 8 | 103,000,001 | 145,000,000 | 42 | 0.266986587 | 5 | HLAMP | FALSE | 4.9186783 | 5 | HLAMP |
| C84 | 9 | 1,000,001 | 37,000,000 | 36 | -0.094281564 | 1 | HETD | FALSE | 0.9266645 | 1 | HETD |
| C84 | 10 | 1,000,001 | 135,000,000 | 134 | -0.017618192 | 1 | HETD | TRUE | 1.1672996 | 1 | HETD |
| C84 | 11 | 2,000,001 | 23,000,000 | 21 | -0.08541476 | 1 | HETD | FALSE | 1.0131522 | 1 | HETD |
| C84 | 11 | 23,000,001 | 134,000,000 | 111 | -0.015045867 | 1 | HETD | TRUE | 1.2387532 | 1 | HETD |
| C84 | 12 | 1,000,001 | 39,000,000 | 38 | 0.126860503 | 3 | GAIN | FALSE | 3.2506511 | 3 | GAIN |
| C84 | 12 | 53,000,001 | 69,000,000 | 16 | 0.152927117 | 3 | GAIN | FALSE | 3.5488022 | 3 | GAIN |
| C84 | 12 | 39,000,001 | 53,000,000 | 14 | -0.039068182 | 1 | HETD | TRUE | 0.5764003 | 1 | HETD |
| C84 | 12 | 69,000,001 | 132,000,000 | 63 | -0.041086512 | 1 | HETD | TRUE | 0.5212508 | 1 | HETD |
| C84 | 14 | 64,000,001 | 106,000,000 | 42 | 0.04161021 | 3 | GAIN | TRUE | 2.8452773 | 3 | GAIN |
| C84 | 14 | 21,000,001 | 64,000,000 | 43 | -0.070227509 | 1 | HETD | FALSE | 1.1625308 | 1 | HETD |
| C84 | 17 | 1,000,001 | 7,000,000 | 6 | 0.038828694 | 3 | GAIN | TRUE | 2.7649241 | 3 | GAIN |
| C84 | 17 | 26,000,001 | 43,000,000 | 17 | 0.131921477 | 3 | GAIN | FALSE | 3.3081181 | 3 | GAIN |
| C84 | 17 | 43,000,001 | 81,000,000 | 38 | 0.03317333 | 3 | GAIN | TRUE | 2.6020272 | 3 | GAIN |
| C84 | 17 | 7,000,001 | 18,000,000 | 11 | -0.083852321 | 1 | HETD | FALSE | 1.0284476 | 1 | HETD |
| C84 | 18 | 1,000,001 | 20,000,000 | 19 | 0.117791722 | 3 | GAIN | FALSE | 3.1481787 | 3 | GAIN |
| C84 | 18 | 20,000,001 | 78,000,000 | 58 | -0.089360531 | 1 | HETD | FALSE | 0.9745991 | 1 | HETD |
| C84 | 19 | 9,000,001 | 59,000,000 | 50 | 0.043299606 | 3 | GAIN | TRUE | 2.8941568 | 3 | GAIN |
| C84 | 19 | 1,000,001 | 7,000,000 | 6 | -0.095692519 | 1 | HETD | FALSE | 0.9129509 | 1 | HETD |
| C84 | 20 | 1,000,001 | 25,000,000 | 24 | -0.095699733 | 1 | HETD | FALSE | 0.9128808 | 1 | HETD |
| C84 | 22 | 22,000,001 | 50,000,000 | 28 | -0.088396811 | 1 | HETD | FALSE | 0.9840057 | 1 | HETD |
| C85 | 1 | 150,000,001 | 248,000,000 | 98 | 0.061513631 | 3 | GAIN | FALSE | 3.1939038 | 3 | GAIN |
| C85 | 1 | 1,000,001 | 120,000,000 | 119 | -0.015495987 | 1 | HETD | FALSE | 1.2974657 | 1 | HETD |
| C85 | 2 | 1,000,001 | 24,000,000 | 23 | -0.021025838 | 1 | HETD | FALSE | 1.1651436 | 1 | HETD |
| C85 | 2 | 76,000,001 | 141,000,000 | 65 | -0.029615644 | 1 | HETD | FALSE | 0.960604 | 1 | HETD |
| C85 | 2 | 212,000,001 | 243,000,000 | 31 | -0.016886745 | 1 | HETD | FALSE | 1.2641389 | 1 | HETD |
| C85 | 3 | 1,000,001 | 83,000,000 | 82 | -0.021574062 | 1 | HETD | FALSE | 1.1520529 | 1 | HETD |
| C85 | 6 | 28,000,001 | 34,000,000 | 6 | -6.079366862 | 1 | HETD | TRUE | 0.015625 | 1 | HETD |
| C85 | 6 | 75,000,001 | 170,000,000 | 95 | -0.026779305 | 1 | HETD | FALSE | 1.028008 | 1 | HETD |
| C85 | 7 | 1,000,001 | 46,000,000 | 45 | 0.053551397 | 3 | GAIN | FALSE | 2.9931008 | 3 | GAIN |
| C85 | 8 | 1,000,001 | 42,000,000 | 41 | -0.051264726 | 1 | HETD | FALSE | 0.4504683 | 1 | HETD |
| C85 | 11 | 2,000,001 | 134,000,000 | 132 | -0.005006152 | 1 | HETD | FALSE | 1.5498716 | 1 | HETD |
| C85 | 13 | 20,000,001 | 114,000,000 | 94 | -0.036097673 | 1 | HETD | FALSE | 0.8070587 | 1 | HETD |
| C85 | 14 | 21,000,001 | 106,000,000 | 85 | -0.033939401 | 1 | HETD | FALSE | 0.8581069 | 1 | HETD |
| C85 | 16 | 1,000,001 | 90,000,000 | 89 | -0.038737805 | 1 | HETD | FALSE | 0.7447172 | 1 | HETD |
| C85 | 17 | 26,000,001 | 81,000,000 | 55 | 0.035045948 | 3 | GAIN | FALSE | 2.5306626 | 3 | GAIN |
| C85 | 17 | 1,000,001 | 18,000,000 | 17 | -0.032147574 | 1 | HETD | FALSE | 0.9005459 | 1 | HETD |
| C85 | 18 | 1,000,001 | 78,000,000 | 77 | -0.021522125 | 1 | HETD | FALSE | 1.1532929 | 1 | HETD |
| C85 | 20 | 1,000,001 | 61,000,000 | 60 | -0.01714066 | 1 | HETD | FALSE | 1.2580578 | 1 | HETD |
| C85 | X | 4,000,001 | 151,000,000 | 147 | -0.022552209 | 1 | HETD | FALSE | 1.1287088 | 1 | HETD |
| C86 | 1 | 158,000,001 | 193,000,000 | 35 | -0.000380871 | 1 | HETD | TRUE | 1.1452022 | 1 | HETD |
| C86 | 1 | 193,000,001 | 198,000,000 | 5 | -0.232924557 | 1 | HETD | FALSE | 0.9638466 | 1 | HETD |
| C86 | 4 | 2,000,001 | 37,000,000 | 35 | -0.22261073 | 1 | HETD | FALSE | 0.9975957 | 1 | HETD |
| C86 | 4 | 168,000,001 | 191,000,000 | 23 | -0.235504096 | 1 | HETD | FALSE | 0.9554434 | 1 | HETD |
| C86 | 4 | 37,000,001 | 48,000,000 | 11 | 1.178433705 | 5 | HLAMP | FALSE | 8.7718386 | 9 | HLAMP |
| C86 | 5 | 40,000,001 | 159,000,000 | 119 | 0.006482029 | 1 | HETD | TRUE | 1.2507589 | 1 | HETD |
| C86 | 6 | 63,000,001 | 67,000,000 | 4 | 0.328194367 | 3 | GAIN | FALSE | 3.2001729 | 3 | GAIN |
| C86 | 6 | 67,000,001 | 170,000,000 | 103 | -0.222238515 | 1 | HETD | FALSE | 0.9988182 | 1 | HETD |
| C86 | 6 | 28,000,001 | 34,000,000 | 6 | -4.371918526 | 5 | HLAMP | FALSE | 0.015625 | 0 | HOMD |
| C86 | 7 | 1,000,001 | 68,000,000 | 67 | 0.298974021 | 3 | GAIN | FALSE | 3.0610183 | 3 | GAIN |
| C86 | 7 | 117,000,001 | 159,000,000 | 42 | -0.025799715 | 1 | HETD | TRUE | 0.7585859 | 1 | HETD |
| C86 | 8 | 47,000,001 | 145,000,000 | 98 | 0.461705694 | 4 | AMP | FALSE | 3.8731057 | 4 | AMP |
| C86 | 8 | 1,000,001 | 43,000,000 | 42 | -0.226637292 | 1 | HETD | FALSE | 0.9843912 | 1 | HETD |
| C86 | 9 | 1,000,001 | 38,000,000 | 37 | -0.23905024 | 1 | HETD | FALSE | 0.9439159 | 1 | HETD |
| C86 | 10 | 62,000,001 | 135,000,000 | 73 | 0.281878085 | 3 | GAIN | FALSE | 2.9809004 | 3 | GAIN |
| C86 | 10 | 1,000,001 | 20,000,000 | 19 | -0.230248358 | 1 | HETD | FALSE | 0.9725805 | 1 | HETD |
| C86 | 11 | 2,000,001 | 48,000,000 | 46 | 0.130628822 | 3 | GAIN | TRUE | 3.2495438 | 3 | GAIN |
| C86 | 11 | 55,000,001 | 134,000,000 | 79 | -0.069972152 | 1 | HETD | TRUE | 0.1027238 | 1 | HETD |
| C86 | 13 | 20,000,001 | 114,000,000 | 94 | -0.223626388 | 1 | HETD | FALSE | 0.9942615 | 1 | HETD |
| C86 | 14 | 86,000,001 | 106,000,000 | 20 | -0.202597665 | 1 | HETD | FALSE | 1.0637752 | 1 | HETD |
| C86 | 15 | 25,000,001 | 102,000,000 | 77 | 0.007790553 | 1 | HETD | TRUE | 1.270942 | 1 | HETD |
| C86 | 17 | 26,000,001 | 81,000,000 | 55 | 0.104123629 | 3 | GAIN | TRUE | 2.8082476 | 3 | GAIN |
| C86 | 17 | 1,000,001 | 18,000,000 | 17 | -0.200651469 | 1 | HETD | FALSE | 1.07026 | 1 | HETD |
| C86 | 18 | 1,000,001 | 20,000,000 | 19 | 0.163998359 | 3 | GAIN | TRUE | 3.8167813 | 3 | GAIN |
| C86 | 18 | 34,000,001 | 37,000,000 | 3 | 0.276364105 | 3 | GAIN | FALSE | 2.9552617 | 3 | GAIN |
| C86 | 18 | 20,000,001 | 34,000,000 | 14 | -0.005037891 | 1 | HETD | TRUE | 1.0738591 | 1 | HETD |
| C86 | 18 | 37,000,001 | 78,000,000 | 41 | -0.243238384 | 1 | HETD | FALSE | 0.9303378 | 1 | HETD |
| C86 | 19 | 10,000,001 | 24,000,000 | 14 | 0.14369964 | 3 | GAIN | TRUE | 3.4701684 | 3 | GAIN |
| C86 | 19 | 35,000,001 | 46,000,000 | 11 | 0.106483878 | 3 | GAIN | TRUE | 2.8472164 | 3 | GAIN |
| C86 | 19 | 1,000,001 | 10,000,000 | 9 | -0.210194897 | 1 | HETD | FALSE | 1.0385444 | 1 | HETD |
| C86 | 19 | 28,000,001 | 35,000,000 | 7 | -0.012508166 | 1 | HETD | TRUE | 0.9598983 | 1 | HETD |
| C86 | 19 | 46,000,001 | 59,000,000 | 13 | 0.023955951 | 1 | HETD | TRUE | 1.5217991 | 1 | HETD |
| C86 | 20 | 1,000,001 | 61,000,000 | 60 | -0.224686768 | 1 | HETD | FALSE | 0.990783 | 1 | HETD |
| C86 | 21 | 16,000,001 | 48,000,000 | 32 | -0.183838301 | 1 | HETD | FALSE | 1.1266481 | 1 | HETD |
| C86 | 22 | 22,000,001 | 50,000,000 | 28 | -0.1950365 | 1 | HETD | FALSE | 1.0890185 | 1 | HETD |
| C86 | X | 4,000,001 | 151,000,000 | 147 | -0.006917723 | 1 | HETD | TRUE | 0.5225631 | 1 | HETD |
| C87 | 1 | 150,000,001 | 248,000,000 | 98 | 0.05420061 | 3 | GAIN | FALSE | 3.2540394 | 3 | GAIN |
| C87 | 1 | 1,000,001 | 28,000,000 | 27 | -0.047241049 | 1 | HETD | FALSE | 0.730105 | 1 | HETD |
| C87 | 3 | 1,000,001 | 47,000,000 | 46 | -0.029429536 | 1 | HETD | FALSE | 1.1605208 | 1 | HETD |
| C87 | 5 | 45,000,001 | 128,000,000 | 83 | -0.049057402 | 1 | HETD | FALSE | 0.6865106 | 1 | HETD |
| C87 | 6 | 1,000,001 | 170,000,000 | 169 | -0.046649544 | 1 | HETD | FALSE | 0.7443136 | 1 | HETD |
| C87 | 10 | 1,000,001 | 135,000,000 | 134 | -0.026985611 | 1 | HETD | FALSE | 1.2199941 | 1 | HETD |
| C87 | 13 | 20,000,001 | 27,000,000 | 7 | 0.050769881 | 3 | GAIN | FALSE | 3.1657495 | 3 | GAIN |
| C87 | 13 | 27,000,001 | 114,000,000 | 87 | -0.023194208 | 1 | HETD | FALSE | 1.3124579 | 1 | HETD |
| C87 | 16 | 1,000,001 | 90,000,000 | 89 | 0.030876559 | 3 | GAIN | FALSE | 2.6579123 | 3 | GAIN |
| C87 | 19 | 1,000,001 | 59,000,000 | 58 | 0.045081335 | 3 | GAIN | FALSE | 3.0198165 | 3 | GAIN |
| C87 | X | 127,000,001 | 151,000,000 | 24 | 0.038742133 | 3 | GAIN | FALSE | 1.4289341 | 3 | GAIN |
| C87 | X | 4,000,001 | 58,000,000 | 54 | -0.057002324 | 1 | HETD | FALSE | 0.2482339 | 1 | HETD |
| C88 | 1 | 150,000,001 | 178,000,000 | 28 | 0.047376662 | 3 | GAIN | FALSE | 3.0918588 | 3 | GAIN |
| C88 | 4 | 2,000,001 | 28,000,000 | 26 | 0.044766827 | 3 | GAIN | FALSE | 3.036959 | 3 | GAIN |
| C88 | 4 | 178,000,001 | 191,000,000 | 13 | 0.038996047 | 3 | GAIN | FALSE | 2.9159184 | 3 | GAIN |
| C88 | 5 | 1,000,001 | 63,000,000 | 62 | 0.039498105 | 3 | GAIN | FALSE | 2.9264297 | 3 | GAIN |
| C88 | 6 | 28,000,001 | 34,000,000 | 6 | -4.252261399 | 1 | HETD | TRUE | 0.015625 | 1 | HETD |
| C88 | 12 | 114,000,001 | 132,000,000 | 18 | 0.03779861 | 3 | GAIN | FALSE | 2.8908631 | 3 | GAIN |
| C88 | 13 | 20,000,001 | 28,000,000 | 8 | 0.086192373 | 3 | GAIN | FALSE | 3.9202078 | 3 | GAIN |
| C88 | 13 | 79,000,001 | 114,000,000 | 35 | 0.058971602 | 3 | GAIN | FALSE | 3.3369711 | 3 | GAIN |
| C88 | 16 | 1,000,001 | 90,000,000 | 89 | 0.023052824 | 3 | GAIN | FALSE | 2.5840192 | 3 | GAIN |
| C88 | 17 | 55,000,001 | 81,000,000 | 26 | 0.031473835 | 3 | GAIN | FALSE | 2.7588671 | 3 | GAIN |
| C88 | 18 | 1,000,001 | 20,000,000 | 19 | 0.053450409 | 3 | GAIN | FALSE | 3.2200096 | 3 | GAIN |
| C88 | 19 | 1,000,001 | 59,000,000 | 58 | 0.041891536 | 3 | GAIN | FALSE | 2.97659 | 3 | GAIN |
| C88 | 20 | 1,000,001 | 61,000,000 | 60 | 0.051173836 | 3 | GAIN | FALSE | 3.1719126 | 3 | GAIN |
| C88 | 21 | 16,000,001 | 48,000,000 | 32 | 0.027676662 | 3 | GAIN | FALSE | 2.6798989 | 3 | GAIN |
| C88 | X | 94,000,001 | 151,000,000 | 57 | 0.032783802 | 3 | GAIN | FALSE | 2.7861582 | 3 | GAIN |
| C89 | 6 | 28,000,001 | 34,000,000 | 6 | -5.228410504 | 1 | HETD | FALSE | 0.015625 | 1 | HETD |
| C90 | 19 | 1,000,001 | 59,000,000 | 58 | 0.056165561 | 3 | GAIN | FALSE | 3.0673037 | 3 | GAIN |
| C91 | 1 | 150,000,001 | 159,000,000 | 9 | 0.49515025 | 4 | AMP | FALSE | 4.2508004 | 4 | AMP |
| C91 | 1 | 1,000,001 | 7,000,000 | 6 | 0.172634331 | 3 | GAIN | TRUE | 3.1715731 | 3 | GAIN |
| C91 | 1 | 159,000,001 | 184,000,000 | 25 | 0.114472081 | 3 | GAIN | TRUE | 2.784738 | 3 | GAIN |
| C91 | 2 | 1,000,001 | 27,000,000 | 26 | -0.313882832 | 1 | HETD | FALSE | 0.9871205 | 1 | HETD |
| C91 | 3 | 35,000,001 | 43,000,000 | 8 | 0.236814063 | 3 | GAIN | FALSE | 3.0042237 | 3 | GAIN |
| C91 | 3 | 1,000,001 | 8,000,000 | 7 | -0.383937218 | 1 | HETD | FALSE | 0.7814236 | 1 | HETD |
| C91 | 3 | 24,000,001 | 35,000,000 | 11 | -0.320338892 | 1 | HETD | FALSE | 0.9677433 | 1 | HETD |
| C91 | 4 | 73,000,001 | 116,000,000 | 43 | 0.131715172 | 3 | GAIN | TRUE | 2.8977993 | 3 | GAIN |
| C91 | 4 | 133,000,001 | 148,000,000 | 15 | 0.289440693 | 3 | GAIN | FALSE | 3.2403917 | 3 | GAIN |
| C91 | 4 | 116,000,001 | 132,000,000 | 16 | -0.412185187 | 1 | HETD | FALSE | 0.7012654 | 1 | HETD |
| C91 | 4 | 176,000,001 | 191,000,000 | 15 | -0.247000933 | 1 | HETD | FALSE | 1.1930451 | 1 | HETD |
| C91 | 5 | 1,000,001 | 128,000,000 | 127 | -0.315200013 | 1 | HETD | FALSE | 0.9831601 | 1 | HETD |
| C91 | 5 | 144,000,001 | 154,000,000 | 10 | -0.348435378 | 1 | HETD | FALSE | 0.8844174 | 1 | HETD |
| C91 | 6 | 1,000,001 | 28,000,000 | 27 | 0.13452987 | 3 | GAIN | TRUE | 2.9163836 | 3 | GAIN |
| C91 | 6 | 34,000,001 | 170,000,000 | 136 | 0.141486545 | 3 | GAIN | TRUE | 2.9624717 | 3 | GAIN |
| C91 | 6 | 28,000,001 | 34,000,000 | 6 | -5.434718781 | 1 | HETD | TRUE | 0.015625 | 1 | HETD |
| C91 | 7 | 1,000,001 | 6,000,000 | 5 | 0.16631677 | 3 | GAIN | TRUE | 3.1287963 | 3 | GAIN |
| C91 | 8 | 34,000,001 | 43,000,000 | 9 | 0.129031759 | 3 | GAIN | TRUE | 2.8801155 | 3 | GAIN |
| C91 | 8 | 47,000,001 | 145,000,000 | 98 | 0.23174299 | 3 | GAIN | FALSE | 2.9819185 | 3 | GAIN |
| C91 | 8 | 1,000,001 | 34,000,000 | 33 | -0.251916195 | 1 | HETD | TRUE | 0.6758083 | 1 | HETD |
| C91 | 9 | 1,000,001 | 141,000,000 | 140 | -0.302354256 | 1 | HETD | FALSE | 1.0219387 | 1 | HETD |
| C91 | 10 | 43,000,001 | 135,000,000 | 92 | -0.302647123 | 1 | HETD | FALSE | 1.0210507 | 1 | HETD |
| C91 | 11 | 10,000,001 | 134,000,000 | 124 | 0.162939421 | 3 | GAIN | TRUE | 3.1060046 | 3 | GAIN |
| C91 | 11 | 2,000,001 | 10,000,000 | 8 | -0.322974696 | 1 | HETD | FALSE | 0.9598572 | 1 | HETD |
| C91 | 12 | 76,000,001 | 89,000,000 | 13 | -0.351848795 | 1 | HETD | FALSE | 0.8744043 | 1 | HETD |
| C91 | 12 | 1,000,001 | 32,000,000 | 31 | 1.028759865 | 5 | HLAMP | FALSE | 7.653705 | 8 | HLAMP |
| C91 | 13 | 20,000,001 | 76,000,000 | 56 | 0.163150054 | 3 | GAIN | TRUE | 3.1074245 | 3 | GAIN |
| C91 | 13 | 76,000,001 | 104,000,000 | 28 | 0.244435821 | 3 | GAIN | FALSE | 3.0378961 | 3 | GAIN |
| C91 | 13 | 108,000,001 | 114,000,000 | 6 | 0.190695431 | 3 | GAIN | FALSE | 2.8042267 | 3 | GAIN |
| C91 | 13 | 104,000,001 | 108,000,000 | 4 | -0.424657285 | 1 | HETD | FALSE | 0.6663702 | 1 | HETD |
| C91 | 15 | 25,000,001 | 76,000,000 | 51 | 0.125547651 | 3 | GAIN | TRUE | 2.8572041 | 3 | GAIN |
| C91 | 15 | 76,000,001 | 102,000,000 | 26 | 0.207419151 | 3 | GAIN | FALSE | 2.8760127 | 3 | GAIN |
| C91 | 16 | 72,000,001 | 79,000,000 | 7 | 0.52757915 | 4 | AMP | FALSE | 4.4236465 | 4 | AMP |
| C91 | 16 | 58,000,001 | 72,000,000 | 14 | 0.165439611 | 3 | GAIN | TRUE | 3.1228717 | 3 | GAIN |
| C91 | 16 | 79,000,001 | 90,000,000 | 11 | -0.330270405 | 1 | HETD | FALSE | 0.9381038 | 1 | HETD |
| C91 | 17 | 1,000,001 | 18,000,000 | 17 | -0.285105394 | 1 | HETD | FALSE | 1.0745554 | 1 | HETD |
| C91 | 19 | 28,000,001 | 59,000,000 | 31 | 0.459391611 | 4 | AMP | FALSE | 4.0646573 | 4 | AMP |
| C91 | 19 | 1,000,001 | 24,000,000 | 23 | -0.227308145 | 1 | HETD | FALSE | 1.2555194 | 1 | HETD |
| C91 | 20 | 1,000,001 | 25,000,000 | 24 | 0.144191599 | 3 | GAIN | TRUE | 2.9804528 | 3 | GAIN |
| C91 | 20 | 30,000,001 | 61,000,000 | 31 | 0.233208768 | 3 | GAIN | FALSE | 2.9883577 | 3 | GAIN |
| C91 | 21 | 16,000,001 | 30,000,000 | 14 | 0.223298896 | 3 | GAIN | FALSE | 2.9449505 | 3 | GAIN |
| C91 | 21 | 36,000,001 | 48,000,000 | 12 | -0.356866488 | 1 | HETD | FALSE | 0.8597281 | 1 | HETD |
| C91 | 22 | 22,000,001 | 50,000,000 | 28 | -0.312647885 | 1 | HETD | FALSE | 0.9908369 | 1 | HETD |
| C91 | X | 4,000,001 | 151,000,000 | 147 | -0.822219315 | 1 | HETD | TRUE | 0.015625 | 1 | HETD |
| C92 | 7 | 1,000,001 | 100,000,000 | 99 | 0.040179057 | 3 | GAIN | FALSE | 2.802098 | 3 | GAIN |
| C92 | 12 | 1,000,001 | 43,000,000 | 42 | 0.078928403 | 3 | GAIN | FALSE | 3.5837761 | 3 | GAIN |
| C93 | 2 | 76,000,001 | 243,000,000 | 167 | 0.106377119 | 3 | GAIN | TRUE | 3.0488597 | 3 | GAIN |
| C93 | 3 | 94,000,001 | 197,000,000 | 103 | 0.250221028 | 4 | AMP | FALSE | 3.7093589 | 4 | AMP |
| C93 | 3 | 1,000,001 | 90,000,000 | 89 | -0.064292262 | 1 | HETD | TRUE | 0.8432194 | 1 | HETD |
| C93 | 4 | 2,000,001 | 191,000,000 | 189 | -0.039687098 | 1 | HETD | TRUE | 1.1453345 | 1 | HETD |
| C93 | 5 | 50,000,001 | 82,000,000 | 32 | -0.03348928 | 1 | HETD | TRUE | 1.2222505 | 1 | HETD |
| C93 | 5 | 82,000,001 | 180,000,000 | 98 | -0.140573319 | 1 | HETD | FALSE | 0.8712301 | 1 | HETD |
| C93 | 5 | 1,000,001 | 46,000,000 | 45 | 0.421059204 | 5 | HLAMP | FALSE | 5.2129495 | 5 | HLAMP |
| C93 | 6 | 1,000,001 | 57,000,000 | 56 | 0.094933662 | 3 | GAIN | TRUE | 2.8926708 | 3 | GAIN |
| C93 | 6 | 63,000,001 | 170,000,000 | 107 | -0.116059045 | 1 | HETD | FALSE | 1.0275631 | 1 | HETD |
| C93 | 7 | 1,000,001 | 39,000,000 | 38 | -0.116051832 | 1 | HETD | FALSE | 1.0276095 | 1 | HETD |
| C93 | 7 | 144,000,001 | 159,000,000 | 15 | -0.08837517 | 1 | HETD | FALSE | 1.2073327 | 1 | HETD |
| C93 | 8 | 1,000,001 | 43,000,000 | 42 | -0.143034557 | 1 | HETD | FALSE | 0.8556805 | 1 | HETD |
| C93 | 8 | 126,000,001 | 133,000,000 | 7 | 0.84834508 | 5 | HLAMP | FALSE | 9.8540643 | 10 | HLAMP |
| C93 | 9 | 1,000,001 | 141,000,000 | 140 | -0.12591121 | 1 | HETD | FALSE | 0.9644142 | 1 | HETD |
| C93 | 10 | 1,000,001 | 37,000,000 | 36 | 0.313698167 | 4 | AMP | FALSE | 4.2473609 | 4 | AMP |
| C93 | 10 | 43,000,001 | 135,000,000 | 92 | -0.062079168 | 1 | HETD | TRUE | 0.8701825 | 1 | HETD |
| C93 | 12 | 1,000,001 | 39,000,000 | 38 | 0.228775366 | 4 | AMP | FALSE | 3.5328799 | 4 | AMP |
| C93 | 13 | 20,000,001 | 114,000,000 | 94 | -0.066300493 | 1 | HETD | TRUE | 0.8187879 | 1 | HETD |
| C93 | 14 | 21,000,001 | 28,000,000 | 7 | -0.069076239 | 1 | HETD | TRUE | 0.7850751 | 1 | HETD |
| C93 | 14 | 56,000,001 | 106,000,000 | 50 | -0.095919022 | 1 | HETD | FALSE | 1.1580031 | 1 | HETD |
| C93 | 15 | 25,000,001 | 102,000,000 | 77 | -0.021493271 | 1 | HETD | TRUE | 1.372065 | 1 | HETD |
| C93 | 16 | 1,000,001 | 48,000,000 | 47 | -0.093430374 | 1 | HETD | FALSE | 1.174248 | 1 | HETD |
| C93 | 17 | 26,000,001 | 44,000,000 | 18 | 0.201635387 | 3 | GAIN | FALSE | 3.3132698 | 3 | GAIN |
| C93 | 17 | 45,000,001 | 70,000,000 | 25 | 0.098484134 | 3 | GAIN | TRUE | 2.9409979 | 3 | GAIN |
| C93 | 17 | 1,000,001 | 18,000,000 | 17 | -0.104396299 | 1 | HETD | FALSE | 1.1028767 | 1 | HETD |
| C93 | 18 | 43,000,001 | 72,000,000 | 29 | -0.127591949 | 1 | HETD | FALSE | 0.9536842 | 1 | HETD |
| C93 | 19 | 21,000,001 | 59,000,000 | 38 | 0.175816917 | 3 | GAIN | FALSE | 3.1081527 | 3 | GAIN |
| C93 | 19 | 1,000,001 | 20,000,000 | 19 | -0.045733433 | 1 | HETD | TRUE | 1.0706162 | 1 | HETD |
| C93 | 20 | 1,000,001 | 61,000,000 | 60 | 0.207165237 | 3 | GAIN | FALSE | 3.3576815 | 3 | GAIN |
| C93 | 21 | 16,000,001 | 33,000,000 | 17 | -0.02853218 | 1 | HETD | TRUE | 1.2840072 | 1 | HETD |
| C93 | 21 | 34,000,001 | 48,000,000 | 14 | -0.156018813 | 1 | HETD | FALSE | 0.7740862 | 1 | HETD |
| C93 | 22 | 22,000,001 | 50,000,000 | 28 | 0.122732953 | 3 | GAIN | TRUE | 3.2742582 | 3 | GAIN |
| C93 | X | 102,000,001 | 134,000,000 | 32 | -0.039026344 | 1 | HETD | TRUE | 0.5767594 | 1 | HETD |
| C94 | 1 | 36,000,001 | 248,000,000 | 212 | 0.07008901 | 3 | GAIN | FALSE | 3.1588264 | 3 | GAIN |
| C94 | 1 | 1,000,001 | 36,000,000 | 35 | -0.217569954 | 1 | HETD | FALSE | 0.9403789 | 1 | HETD |
| C94 | 2 | 1,000,001 | 243,000,000 | 242 | 0.044833191 | 3 | GAIN | FALSE | 2.9458582 | 3 | GAIN |
| C94 | 3 | 130,000,001 | 197,000,000 | 67 | 0.14993641 | 4 | AMP | FALSE | 3.8571931 | 4 | AMP |
| C94 | 3 | 1,000,001 | 20,000,000 | 19 | 0.2553022 | 5 | HLAMP | FALSE | 4.8399379 | 5 | HLAMP |
| C94 | 4 | 2,000,001 | 191,000,000 | 189 | -0.205374853 | 1 | HETD | FALSE | 1.0257237 | 1 | HETD |
| C94 | 5 | 75,000,001 | 180,000,000 | 105 | 0.061416971 | 3 | GAIN | FALSE | 3.0852793 | 3 | GAIN |
| C94 | 5 | 50,000,001 | 75,000,000 | 25 | -0.249757923 | 1 | HETD | FALSE | 0.7185527 | 1 | HETD |
| C94 | 5 | 1,000,001 | 46,000,000 | 45 | 0.216889002 | 5 | HLAMP | FALSE | 4.4733187 | 4 | AMP |
| C94 | 6 | 1,000,001 | 23,000,000 | 22 | -0.217532444 | 1 | HETD | FALSE | 0.9406403 | 1 | HETD |
| C94 | 6 | 79,000,001 | 170,000,000 | 91 | -0.209639459 | 1 | HETD | FALSE | 0.9957967 | 1 | HETD |
| C94 | 6 | 23,000,001 | 79,000,000 | 56 | 0.260886872 | 5 | HLAMP | FALSE | 4.894056 | 5 | HLAMP |
| C94 | 7 | 1,000,001 | 159,000,000 | 158 | 0.134783784 | 4 | AMP | FALSE | 3.7216758 | 4 | AMP |
| C94 | 8 | 1,000,001 | 32,000,000 | 31 | -0.226862352 | 1 | HETD | FALSE | 0.8758306 | 1 | HETD |
| C94 | 8 | 32,000,001 | 145,000,000 | 113 | 0.324107212 | 5 | HLAMP | FALSE | 5.5215248 | 6 | HLAMP |
| C94 | 9 | 1,000,001 | 8,000,000 | 7 | 0.124291063 | 4 | AMP | FALSE | 3.6286649 | 4 | AMP |
| C94 | 9 | 8,000,001 | 141,000,000 | 133 | -0.203675358 | 1 | HETD | FALSE | 1.0376747 | 1 | HETD |
| C94 | 11 | 2,000,001 | 134,000,000 | 132 | -0.001014215 | 3 | GAIN | TRUE | 3.0586502 | 3 | GAIN |
| C94 | 12 | 1,000,001 | 132,000,000 | 131 | -0.005146093 | 3 | GAIN | TRUE | 2.9964522 | 3 | GAIN |
| C94 | 13 | 64,000,001 | 114,000,000 | 50 | -0.223955322 | 1 | HETD | FALSE | 0.8959792 | 1 | HETD |
| C94 | 14 | 21,000,001 | 84,000,000 | 63 | 0.151361793 | 4 | AMP | FALSE | 3.8700143 | 4 | AMP |
| C94 | 14 | 84,000,001 | 106,000,000 | 22 | -0.232703825 | 1 | HETD | FALSE | 0.8354659 | 1 | HETD |
| C94 | 16 | 47,000,001 | 90,000,000 | 43 | -0.200319649 | 1 | HETD | FALSE | 1.0613136 | 1 | HETD |
| C94 | 16 | 1,000,001 | 32,000,000 | 31 | 0.235843129 | 5 | HLAMP | FALSE | 4.6529988 | 5 | HLAMP |
| C94 | 17 | 1,000,001 | 18,000,000 | 17 | -0.162207974 | 1 | HETD | FALSE | 1.3336809 | 1 | HETD |
| C94 | 17 | 26,000,001 | 81,000,000 | 55 | 0.309139251 | 5 | HLAMP | FALSE | 5.3704723 | 5 | HLAMP |
| C94 | 18 | 1,000,001 | 20,000,000 | 19 | 0.124948932 | 4 | AMP | FALSE | 3.6344766 | 4 | AMP |
| C94 | 18 | 20,000,001 | 41,000,000 | 21 | 0.062245078 | 3 | GAIN | FALSE | 3.0922833 | 3 | GAIN |
| C94 | 18 | 41,000,001 | 78,000,000 | 37 | -0.009065896 | 3 | GAIN | TRUE | 2.937611 | 3 | GAIN |
| C94 | 19 | 1,000,001 | 59,000,000 | 58 | 0.055449984 | 3 | GAIN | FALSE | 3.0349297 | 3 | GAIN |
| C94 | 20 | 19,000,001 | 61,000,000 | 42 | 0.063211683 | 3 | GAIN | FALSE | 3.1004639 | 3 | GAIN |
| C94 | 20 | 1,000,001 | 19,000,000 | 18 | -0.233044301 | 1 | HETD | FALSE | 0.8331182 | 1 | HETD |
| C94 | 21 | 16,000,001 | 48,000,000 | 32 | 0.098831824 | 3 | GAIN | FALSE | 3.4057779 | 3 | GAIN |
| C94 | 22 | 22,000,001 | 50,000,000 | 28 | 0.051129112 | 3 | GAIN | FALSE | 2.9985999 | 3 | GAIN |
| C94 | X | 4,000,001 | 58,000,000 | 54 | 0.08153391 | 3 | GAIN | FALSE | 3.2565692 | 3 | GAIN |
| C94 | X | 129,000,001 | 151,000,000 | 22 | 0.090777257 | 3 | GAIN | FALSE | 3.3360781 | 3 | GAIN |
| C94 | X | 77,000,001 | 129,000,000 | 52 | -0.188343838 | 1 | HETD | FALSE | 1.1461254 | 1 | HETD |
| C94 | X | 64,000,001 | 74,000,000 | 10 | 0.288479858 | 5 | HLAMP | FALSE | 5.1645412 | 5 | HLAMP |
| C95 | 1 | 150,000,001 | 204,000,000 | 54 | 0.228424791 | 4 | AMP | FALSE | 4.2908238 | 4 | AMP |
| C95 | 1 | 236,000,001 | 248,000,000 | 12 | 0.232915901 | 4 | AMP | FALSE | 4.3347487 | 4 | AMP |
| C95 | 1 | 14,000,001 | 51,000,000 | 37 | 0.043112056 | 3 | GAIN | FALSE | 2.5926092 | 3 | GAIN |
| C95 | 1 | 204,000,001 | 235,000,000 | 31 | 0.079029392 | 3 | GAIN | FALSE | 2.9049422 | 3 | GAIN |
| C95 | 1 | 105,000,001 | 120,000,000 | 15 | -0.202757804 | 1 | HETD | FALSE | 0.6511867 | 1 | HETD |
| C95 | 2 | 189,000,001 | 213,000,000 | 24 | 0.180968781 | 4 | AMP | FALSE | 3.8349453 | 4 | AMP |
| C95 | 2 | 1,000,001 | 43,000,000 | 42 | 0.066024939 | 3 | GAIN | FALSE | 2.790958 | 3 | GAIN |
| C95 | 2 | 174,000,001 | 189,000,000 | 15 | 0.098424984 | 3 | GAIN | FALSE | 3.0768653 | 3 | GAIN |
| C95 | 2 | 213,000,001 | 243,000,000 | 30 | 0.106228521 | 3 | GAIN | FALSE | 3.1466908 | 3 | GAIN |
| C95 | 2 | 129,000,001 | 174,000,000 | 45 | -0.115694043 | 1 | HETD | TRUE | 0.8321707 | 1 | HETD |
| C95 | 3 | 94,000,001 | 104,000,000 | 10 | 0.112463849 | 3 | GAIN | FALSE | 3.2027561 | 3 | GAIN |
| C95 | 3 | 1,000,001 | 90,000,000 | 89 | -0.101422904 | 1 | HETD | TRUE | 1.0165431 | 1 | HETD |
| C95 | 3 | 122,000,001 | 135,000,000 | 13 | -0.127508273 | 1 | HETD | FALSE | 1.2106369 | 1 | HETD |
| C95 | 3 | 104,000,001 | 122,000,000 | 18 | 0.280755668 | 5 | HLAMP | FALSE | 4.8112264 | 5 | HLAMP |
| C95 | 3 | 135,000,001 | 197,000,000 | 62 | 0.305629174 | 5 | HLAMP | FALSE | 5.0652772 | 5 | HLAMP |
| C95 | 5 | 1,000,001 | 51,000,000 | 50 | 0.194561853 | 4 | AMP | FALSE | 3.9639961 | 4 | AMP |
| C95 | 6 | 124,000,001 | 150,000,000 | 26 | -0.085258949 | 1 | HETD | TRUE | 1.2275839 | 1 | HETD |
| C95 | 7 | 18,000,001 | 44,000,000 | 26 | 0.175040747 | 4 | AMP | FALSE | 3.779045 | 4 | AMP |
| C95 | 7 | 1,000,001 | 18,000,000 | 17 | 0.054290057 | 3 | GAIN | FALSE | 2.6889797 | 3 | GAIN |
| C95 | 8 | 1,000,001 | 39,000,000 | 38 | -0.098677455 | 1 | HETD | TRUE | 1.0522219 | 1 | HETD |
| C95 | 9 | 71,000,001 | 80,000,000 | 9 | 0.105696167 | 3 | GAIN | FALSE | 3.1419153 | 3 | GAIN |
| C95 | 9 | 118,000,001 | 141,000,000 | 23 | 0.091989121 | 3 | GAIN | FALSE | 3.0195612 | 3 | GAIN |
| C95 | 9 | 1,000,001 | 38,000,000 | 37 | -0.106365577 | 1 | HETD | TRUE | 0.952481 | 1 | HETD |
| C95 | 9 | 80,000,001 | 118,000,000 | 38 | -0.123493253 | 1 | HETD | TRUE | 0.7321789 | 1 | HETD |
| C95 | 10 | 12,000,001 | 31,000,000 | 19 | 0.081063592 | 3 | GAIN | FALSE | 2.9228651 | 3 | GAIN |
| C95 | 11 | 102,000,001 | 134,000,000 | 32 | 0.227964571 | 4 | AMP | FALSE | 4.2863303 | 4 | AMP |
| C95 | 11 | 2,000,001 | 102,000,000 | 100 | 0.080381197 | 3 | GAIN | FALSE | 2.9168499 | 3 | GAIN |
| C95 | 12 | 20,000,001 | 39,000,000 | 19 | 0.180824511 | 4 | AMP | FALSE | 3.8335821 | 4 | AMP |
| C95 | 12 | 1,000,001 | 20,000,000 | 19 | 0.106974395 | 3 | GAIN | FALSE | 3.1533846 | 3 | GAIN |
| C95 | 12 | 39,000,001 | 80,000,000 | 41 | 0.117038635 | 3 | GAIN | FALSE | 3.244045 | 3 | GAIN |
| C95 | 12 | 80,000,001 | 87,000,000 | 7 | 0.265627568 | 5 | HLAMP | FALSE | 4.6588409 | 5 | HLAMP |
| C95 | 13 | 92,000,001 | 114,000,000 | 22 | 0.093912234 | 3 | GAIN | FALSE | 3.0366576 | 3 | GAIN |
| C95 | 14 | 75,000,001 | 106,000,000 | 31 | -0.101473398 | 1 | HETD | TRUE | 1.0158876 | 1 | HETD |
| C95 | 15 | 51,000,001 | 87,000,000 | 36 | -0.082571208 | 1 | HETD | TRUE | 1.2629056 | 1 | HETD |
| C95 | 17 | 26,000,001 | 81,000,000 | 55 | 0.055540874 | 3 | GAIN | FALSE | 2.6998101 | 3 | GAIN |
| C95 | 19 | 45,000,001 | 59,000,000 | 14 | 0.191056104 | 4 | AMP | FALSE | 3.9305965 | 4 | AMP |
| C95 | 19 | 14,000,001 | 24,000,000 | 10 | 0.090197294 | 3 | GAIN | FALSE | 3.0036525 | 3 | GAIN |
| C95 | 19 | 28,000,001 | 45,000,000 | 17 | 0.321168442 | 5 | HLAMP | FALSE | 5.2262281 | 5 | HLAMP |
| C95 | 20 | 21,000,001 | 44,000,000 | 23 | 0.173019531 | 4 | AMP | FALSE | 3.7600377 | 4 | AMP |
| C95 | 20 | 9,000,001 | 15,000,000 | 6 | 0.059287553 | 3 | GAIN | FALSE | 2.7323075 | 3 | GAIN |
| C95 | 20 | 44,000,001 | 61,000,000 | 17 | 0.125922751 | 3 | GAIN | FALSE | 3.3246019 | 3 | GAIN |
| C95 | 20 | 1,000,001 | 9,000,000 | 8 | -0.120870433 | 1 | HETD | TRUE | 0.7657451 | 1 | HETD |
| C95 | 21 | 34,000,001 | 48,000,000 | 14 | -0.090403599 | 1 | HETD | TRUE | 1.1601573 | 1 | HETD |
| C95 | 22 | 34,000,001 | 42,000,000 | 8 | 0.142796512 | 3 | GAIN | FALSE | 3.4789776 | 3 | GAIN |
| C95 | 22 | 22,000,001 | 34,000,000 | 12 | -0.12047802 | 1 | HETD | TRUE | 0.7707724 | 1 | HETD |
| C95 | X | 4,000,001 | 151,000,000 | 147 | 0.064639951 | 3 | GAIN | FALSE | 2.778879 | 3 | GAIN |
| C96 | 1 | 1,000,001 | 11,000,000 | 10 | 0.092724896 | 3 | GAIN | FALSE | 3.1570545 | 3 | GAIN |
| C96 | 1 | 109,000,001 | 196,000,000 | 87 | 0.06947875 | 3 | GAIN | FALSE | 2.8437521 | 3 | GAIN |
| C96 | 1 | 207,000,001 | 248,000,000 | 41 | 0.099939814 | 3 | GAIN | FALSE | 3.2553253 | 3 | GAIN |
| C96 | 1 | 196,000,001 | 205,000,000 | 9 | -0.031987434 | 1 | HETD | TRUE | 1.2966973 | 1 | HETD |
| C96 | 2 | 1,000,001 | 21,000,000 | 20 | -0.043482829 | 1 | HETD | TRUE | 1.0814434 | 1 | HETD |
| C96 | 3 | 1,000,001 | 35,000,000 | 34 | 0.059919453 | 3 | GAIN | FALSE | 2.7163733 | 3 | GAIN |
| C96 | 3 | 127,000,001 | 197,000,000 | 70 | 0.089161439 | 3 | GAIN | FALSE | 3.1086994 | 3 | GAIN |
| C96 | 3 | 35,000,001 | 78,000,000 | 43 | -0.05628819 | 1 | HETD | TRUE | 0.8436709 | 1 | HETD |
| C96 | 4 | 96,000,001 | 105,000,000 | 9 | 0.062426857 | 3 | GAIN | FALSE | 2.7497032 | 3 | GAIN |
| C96 | 4 | 2,000,001 | 96,000,000 | 94 | -0.045323707 | 1 | HETD | TRUE | 1.0471315 | 1 | HETD |
| C96 | 4 | 105,000,001 | 132,000,000 | 27 | -0.075774672 | 1 | HETD | FALSE | 0.9963726 | 1 | HETD |
| C96 | 5 | 54,000,001 | 180,000,000 | 126 | -0.084328411 | 1 | HETD | FALSE | 0.8932741 | 1 | HETD |
| C96 | 6 | 87,000,001 | 135,000,000 | 48 | 0.054908973 | 3 | GAIN | FALSE | 2.6499444 | 3 | GAIN |
| C96 | 6 | 1,000,001 | 17,000,000 | 16 | -0.094473442 | 1 | HETD | FALSE | 0.7717854 | 1 | HETD |
| C96 | 7 | 38,000,001 | 137,000,000 | 99 | 0.074907612 | 3 | GAIN | FALSE | 2.916469 | 3 | GAIN |
| C96 | 8 | 1,000,001 | 91,000,000 | 90 | -0.068398172 | 1 | HETD | FALSE | 1.0857741 | 1 | HETD |
| C96 | 9 | 1,000,001 | 30,000,000 | 29 | -0.080584617 | 1 | HETD | FALSE | 0.938323 | 1 | HETD |
| C96 | 9 | 72,000,001 | 141,000,000 | 69 | -0.047522375 | 1 | HETD | TRUE | 1.0062083 | 1 | HETD |
| C96 | 11 | 88,000,001 | 118,000,000 | 30 | 0.10449873 | 3 | GAIN | FALSE | 3.3176739 | 3 | GAIN |
| C96 | 11 | 2,000,001 | 48,000,000 | 46 | -0.076954796 | 1 | HETD | FALSE | 0.9821122 | 1 | HETD |
| C96 | 12 | 118,000,001 | 132,000,000 | 14 | 0.094222413 | 3 | GAIN | FALSE | 3.177411 | 3 | GAIN |
| C96 | 13 | 99,000,001 | 114,000,000 | 15 | -0.095226529 | 1 | HETD | FALSE | 0.762801 | 1 | HETD |
| C96 | 14 | 34,000,001 | 63,000,000 | 29 | 0.088002955 | 3 | GAIN | FALSE | 3.0930048 | 3 | GAIN |
| C96 | 14 | 63,000,001 | 75,000,000 | 12 | -0.095906038 | 1 | HETD | FALSE | 0.7546985 | 1 | HETD |
| C96 | 15 | 81,000,001 | 102,000,000 | 21 | 0.131393451 | 4 | AMP | FALSE | 3.6895281 | 4 | AMP |
| C96 | 15 | 36,000,001 | 72,000,000 | 36 | -0.091077338 | 1 | HETD | FALSE | 0.8123593 | 1 | HETD |
| C96 | 16 | 78,000,001 | 90,000,000 | 12 | -0.089996759 | 1 | HETD | FALSE | 0.8252892 | 1 | HETD |
| C96 | 17 | 28,000,001 | 81,000,000 | 53 | 0.070295316 | 3 | GAIN | FALSE | 2.8546721 | 3 | GAIN |
| C96 | 17 | 1,000,001 | 28,000,000 | 27 | -0.05750871 | 1 | HETD | TRUE | 0.821118 | 1 | HETD |
| C96 | 18 | 1,000,001 | 14,000,000 | 13 | 0.191640396 | 4 | AMP | FALSE | 4.5481104 | 4 | AMP |
| C96 | 18 | 47,000,001 | 78,000,000 | 31 | -0.087363841 | 1 | HETD | FALSE | 0.8568346 | 1 | HETD |
| C96 | 19 | 12,000,001 | 59,000,000 | 47 | 0.067482061 | 3 | GAIN | FALSE | 2.8170762 | 3 | GAIN |
| C96 | 19 | 1,000,001 | 12,000,000 | 11 | -0.048220639 | 1 | HETD | TRUE | 0.9932247 | 1 | HETD |
| C96 | 21 | 34,000,001 | 48,000,000 | 14 | -0.074754686 | 1 | HETD | FALSE | 1.0087074 | 1 | HETD |
| C96 | X | 4,000,001 | 151,000,000 | 147 | -0.036454018 | 1 | HETD | TRUE | 1.2128557 | 1 | HETD |
| C97 | 1 | 1,000,001 | 248,000,000 | 247 | 0.005174947 | 3 | GAIN | FALSE | 2.8135933 | 3 | GAIN |
| C97 | 2 | 17,000,001 | 102,000,000 | 85 | 0.008719649 | 3 | GAIN | FALSE | 2.909428 | 3 | GAIN |
| C97 | 2 | 135,000,001 | 243,000,000 | 108 | 0.013766196 | 3 | GAIN | FALSE | 3.0462736 | 3 | GAIN |
| C97 | 3 | 141,000,001 | 197,000,000 | 56 | 0.014070605 | 3 | GAIN | FALSE | 3.0545434 | 3 | GAIN |
| C97 | 4 | 2,000,001 | 191,000,000 | 189 | 0.014649125 | 3 | GAIN | FALSE | 3.0702649 | 3 | GAIN |
| C97 | 6 | 1,000,001 | 28,000,000 | 27 | -0.006389696 | 3 | GAIN | FALSE | 2.5025634 | 3 | GAIN |
| C97 | 6 | 46,000,001 | 76,000,000 | 30 | 0.009875248 | 3 | GAIN | FALSE | 2.9407218 | 3 | GAIN |
| C97 | 6 | 28,000,001 | 34,000,000 | 6 | -3.307718857 | 1 | HETD | TRUE | 0.015625 | 1 | HETD |
| C97 | 7 | 1,000,001 | 159,000,000 | 158 | 0.021839518 | 3 | GAIN | FALSE | 3.266193 | 3 | GAIN |
| C97 | 8 | 1,000,001 | 145,000,000 | 144 | 0.016008144 | 3 | GAIN | FALSE | 3.1072215 | 3 | GAIN |
| C97 | 9 | 31,000,001 | 141,000,000 | 110 | -0.0029229 | 3 | GAIN | FALSE | 2.595541 | 3 | GAIN |
| C97 | 10 | 26,000,001 | 135,000,000 | 109 | 0.00020342 | 3 | GAIN | FALSE | 2.679579 | 3 | GAIN |
| C97 | 11 | 2,000,001 | 134,000,000 | 132 | 0.004091483 | 3 | GAIN | FALSE | 2.7843477 | 3 | GAIN |
| C97 | 12 | 1,000,001 | 132,000,000 | 131 | 0.019974113 | 3 | GAIN | FALSE | 3.2152695 | 3 | GAIN |
| C97 | 13 | 20,000,001 | 114,000,000 | 94 | 0.014187463 | 3 | GAIN | FALSE | 3.0577186 | 3 | GAIN |
| C97 | 15 | 25,000,001 | 68,000,000 | 43 | 0.018128906 | 3 | GAIN | FALSE | 3.1649621 | 3 | GAIN |
| C97 | 16 | 1,000,001 | 90,000,000 | 89 | 0.014937664 | 3 | GAIN | FALSE | 3.0781084 | 3 | GAIN |
| C97 | 17 | 26,000,001 | 81,000,000 | 55 | 0.020956588 | 3 | GAIN | FALSE | 3.2420818 | 3 | GAIN |
| C97 | 19 | 1,000,001 | 33,000,000 | 32 | 0.023374545 | 3 | GAIN | FALSE | 3.308147 | 3 | GAIN |
| C97 | 19 | 33,000,001 | 59,000,000 | 26 | 0.071903921 | 5 | HLAMP | FALSE | 4.657786 | 5 | HLAMP |
| C97 | 20 | 1,000,001 | 61,000,000 | 60 | -0.000604489 | 3 | GAIN | FALSE | 2.6578443 | 3 | GAIN |
| C97 | 21 | 16,000,001 | 48,000,000 | 32 | 0.016602535 | 3 | GAIN | FALSE | 3.123396 | 3 | GAIN |
| C97 | X | 4,000,001 | 58,000,000 | 54 | 0.012411505 | 3 | GAIN | FALSE | 1.5047459 | 3 | GAIN |
| C97 | X | 118,000,001 | 151,000,000 | 33 | 0.013803706 | 3 | GAIN | FALSE | 1.5236463 | 3 | GAIN |
| C98 | 1 | 1,000,001 | 9,000,000 | 8 | 0.051753799 | 3 | GAIN | FALSE | 3.0863059 | 3 | GAIN |
| C98 | 1 | 32,000,001 | 248,000,000 | 216 | 0.04906173 | 3 | GAIN | FALSE | 3.0527388 | 3 | GAIN |
| C98 | 1 | 9,000,001 | 32,000,000 | 23 | -0.150957838 | 1 | HETD | FALSE | 0.7260576 | 1 | HETD |
| C98 | 2 | 1,000,001 | 243,000,000 | 242 | 0.047891705 | 3 | GAIN | FALSE | 3.0381694 | 3 | GAIN |
| C98 | 4 | 2,000,001 | 191,000,000 | 189 | 0.016909829 | 3 | GAIN | FALSE | 2.6566443 | 3 | GAIN |
| C98 | 6 | 80,000,001 | 127,000,000 | 47 | -0.134638072 | 1 | HETD | FALSE | 0.9040426 | 1 | HETD |
| C98 | 7 | 1,000,001 | 71,000,000 | 70 | 0.207792809 | 5 | HLAMP | FALSE | 5.1429731 | 5 | HLAMP |
| C98 | 8 | 1,000,001 | 145,000,000 | 144 | 0.052746373 | 3 | GAIN | FALSE | 3.0986981 | 3 | GAIN |
| C98 | 9 | 71,000,001 | 133,000,000 | 62 | 0.048014334 | 3 | GAIN | FALSE | 3.0396959 | 3 | GAIN |
| C98 | 9 | 1,000,001 | 38,000,000 | 37 | -0.130546589 | 1 | HETD | FALSE | 0.9489812 | 1 | HETD |
| C98 | 9 | 134,000,001 | 141,000,000 | 7 | -0.144748479 | 1 | HETD | FALSE | 0.7935403 | 1 | HETD |
| C98 | 12 | 1,000,001 | 132,000,000 | 131 | 0.055735638 | 3 | GAIN | FALSE | 3.13607 | 3 | GAIN |
| C98 | 14 | 21,000,001 | 106,000,000 | 85 | -0.131139537 | 1 | HETD | FALSE | 0.9424607 | 1 | HETD |
| C98 | 15 | 25,000,001 | 102,000,000 | 77 | 0.032890562 | 3 | GAIN | FALSE | 2.8524152 | 3 | GAIN |
| C98 | 17 | 26,000,001 | 81,000,000 | 55 | 0.049187245 | 3 | GAIN | FALSE | 3.0543025 | 3 | GAIN |
| C98 | 17 | 1,000,001 | 18,000,000 | 17 | -0.117457017 | 1 | HETD | FALSE | 1.0936092 | 1 | HETD |
| C98 | 18 | 1,000,001 | 78,000,000 | 77 | 0.043453975 | 3 | GAIN | FALSE | 2.9830171 | 3 | GAIN |
| C98 | 19 | 11,000,001 | 59,000,000 | 48 | 0.02459795 | 3 | GAIN | FALSE | 2.7505564 | 3 | GAIN |
| C98 | 20 | 1,000,001 | 61,000,000 | 60 | 0.042087742 | 3 | GAIN | FALSE | 2.9660717 | 3 | GAIN |
| C98 | 21 | 16,000,001 | 48,000,000 | 32 | 0.053285941 | 3 | GAIN | FALSE | 3.105438 | 3 | GAIN |
| C98 | 22 | 22,000,001 | 50,000,000 | 28 | 0.091381747 | 3 | GAIN | FALSE | 3.5877388 | 3 | GAIN |
| C98 | X | 4,000,001 | 151,000,000 | 147 | -0.134651056 | 1 | HETD | FALSE | 0.9039002 | 1 | HETD |
| C99 | 6 | 28,000,001 | 34,000,000 | 6 | -6.1694271 | 1 | HETD | TRUE | 0.015625 | 1 | HETD |
| C99 | 19 | 1,000,001 | 59,000,000 | 58 | 0.037410525 | 3 | GAIN | FALSE | 2.8651064 | 3 | GAIN |
